# Glucocorticoid-Induced Osteoporosis: Why Kids Are Different

**DOI:** 10.3389/fendo.2020.00576

**Published:** 2020-12-16

**Authors:** Leanne M. Ward

**Affiliations:** The Ottawa Pediatric Bone Health Research Group, The Children's Hospital of Eastern Ontario Genetic and Metabolic Bone Disease Clinic, University of Ottawa, Ottawa, ON, Canada

**Keywords:** glucocorticoids, children, fractures, osteoporosis, bone density, risk factors, prevention, treatment

## Abstract

Glucocorticoids (GC) are an important risk factor for bone fragility in children with serious illnesses, largely due to their direct adverse effects on skeletal metabolism. To better appreciate the natural history of fractures in this setting, over a decade ago the Canadian **ST**eroid-associated **O**steoporosis in the **P**ediatric **P**opulation (“**STOPP**”) Consortium launched a 6 year, multi-center observational cohort study in GC-treated children. This study unveiled numerous key clinical-biological principles about GC-induced osteoporosis (GIO), many of which are unique to the growing skeleton. This was important, because most GIO recommendations to date have been guided by adult studies, and therefore do not acknowledge the pediatric-specific principles that inform monitoring, diagnosis and treatment strategies in the young. Some of the most informative observations from the STOPP study were that vertebral fractures are the hallmark of pediatric GIO, they occur early in the GC treatment course, and they are frequently asymptomatic (thereby undetected in the absence of routine monitoring). At the same time, some children have the unique, growth-mediated ability to restore normal vertebral body dimensions following vertebral fractures. This is an important index of recovery, since spontaneous vertebral body reshaping may preclude the need for osteoporosis therapy. Furthermore, we now better understand that children with poor growth, older children with less residual growth potential, and children with ongoing bone health threats have less potential for vertebral body reshaping following spine fractures, which can result in permanent vertebral deformity if treatment is not initiated in a timely fashion. Therefore, pediatric GIO management is now predicated upon early identification of vertebral fractures in those at risk, and timely intervention when there is limited potential for spontaneous recovery. A single, low-trauma long bone fracture can also signal an osteoporotic event, and a need for treatment. Intravenous bisphosphonates are currently the recommended therapy for pediatric GC-induced bone fragility, typically prescribed to children with limited potential for medication-unassisted recovery. It is recognized, however, that even early identification of bone fragility, combined with timely introduction of intravenous bisphosphonate therapy, may not completely rescue the osteoporosis in those with the most aggressive forms, opening the door to novel strategies.

## Introduction

Glucocorticoids (GC) are one of the most potent osteotoxic drugs that are routinely prescribed to treat serious childhood illnesses. Despite major advances in the management of systemic childhood illnesses, GC remain the cornerstone of treatment for many conditions, including leukemia and other cancers, systemic inflammatory or autoimmune disorders, organ transplantation, and some of the neuromuscular disorders such as Duchenne muscular dystrophy (DMD). In the last decade, longitudinal observational cohort studies, including the Canadian **ST**eroid-associated **O**steoporosis in the **P**ediatric **P**opulation (“**STOPP**”) study, have unveiled key clinical-biological principles about GC-induced osteoporosis (GIO) that together inform effective monitoring, diagnosis and treatment strategies. This has been important, since most GC-induced osteoporosis (GIO) recommendations to date have been informed by adult studies and concepts (1), and have thereby fallen short of acknowledging the pediatric-specific principles that guide GIO management in the young.

Given the number and variety of GC-treated disorders in childhood, not to mention the variability in GC prescriptions across and even within diseases, it is important to consider the child's overall health and GC exposure trajectory individually when developing GIO management plans. Since it is beyond the scope of this review article to provide in-depth recommendations on every pediatric GC-treated disease, not to mention on the different clinical scenarios within a given disease, this article instead focuses on key clinical-biological principles that inform the overall approach to pediatric GIO management. In so doing, this article serves as a blueprint for early identification of osteoporosis in any child who is receiving GC therapy, in any clinical context, and provides guidance as to whether osteoporosis therapy is indicated, or not. This article also reviews the evidence for treatment responses to bisphosphonate therapy in those deemed at risk for lack of recovery from GIO, and describes the unmet needs that drive future directions.

## The Effects of Glucocorticoids on the Pediatric Skeleton, and the Impact of the Underlying Disease

There is a long list of GC-treated diseases of childhood. Those that are most frequently associated with skeletal fragility include leukemia and other cancers, systemic inflammatory and autoimmune disorders (such as, but not limited to, inflammatory bowel disease, and rheumatic conditions including systemic lupus erythematosus, systemic-onset juvenile arthritis, juvenile dermatomyositis, systemic vasculitis, and overlap syndromes), renal diseases (e.g., nephrotic syndrome), neuromuscular conditions (e.g., DMD), and organ transplantation. Importantly, many of the underlying diseases themselves carry risk of skeletal fragility, particularly the neuromuscular disorders due to lack of weight-bearing, and the systemic inflammatory and hematological disorders because of the adverse effect of disease-related cytokines on skeletal metabolism (e.g., interleukin [IL] 6 and 1, tumor-necrosis factor-alpha [TNF-α]) (2).

Among the most compelling observations that highlight the cross-talk between cytokines and bone are that severe VF can be one of the presenting signs of childhood leukemia, rheumatic disorders, and inflammatory bowel disease (3–5). The effects of TNF-α on bone are highly similar to the bone formation-blunting effects of GC, with inhibition of osteoblast differentiation, reduction in collagen synthesis by osteoblasts, and promotion of osteoblast apoptosis. Furthermore, IL-1, IL-6, and TNF-α all increase receptor activator of NF-kB ligand (RANKL), which drives osteoclastogenesis. Among boys with DMD, VF are uncommon prior to GC therapy, but long bone fractures can occur before GC initiation because of the adverse effect of the myopathy on bone development. Beyond the loss of normal mechanical strain on bone in DMD from lack of walking, the myopathic process itself is implicated in the bone fragility. The aberrant muscle-bone interactions in DMD involve muscle-derived myokines, bone-derived osteokines, and shared cytokines that catalyze common signaling pathways to incite muscle fibrosis, inflammation, and bone loss (6). Due to the myriad adverse effects of the underlying diseases on bone, the term GIO is often replaced by GC-associated osteoporosis. For the purpose of this article, the conventional term GIO will be used, recognizing that GC are not the only factor leading to bone strength loss, as highlighted in the examples, above.

**Figure 1:**
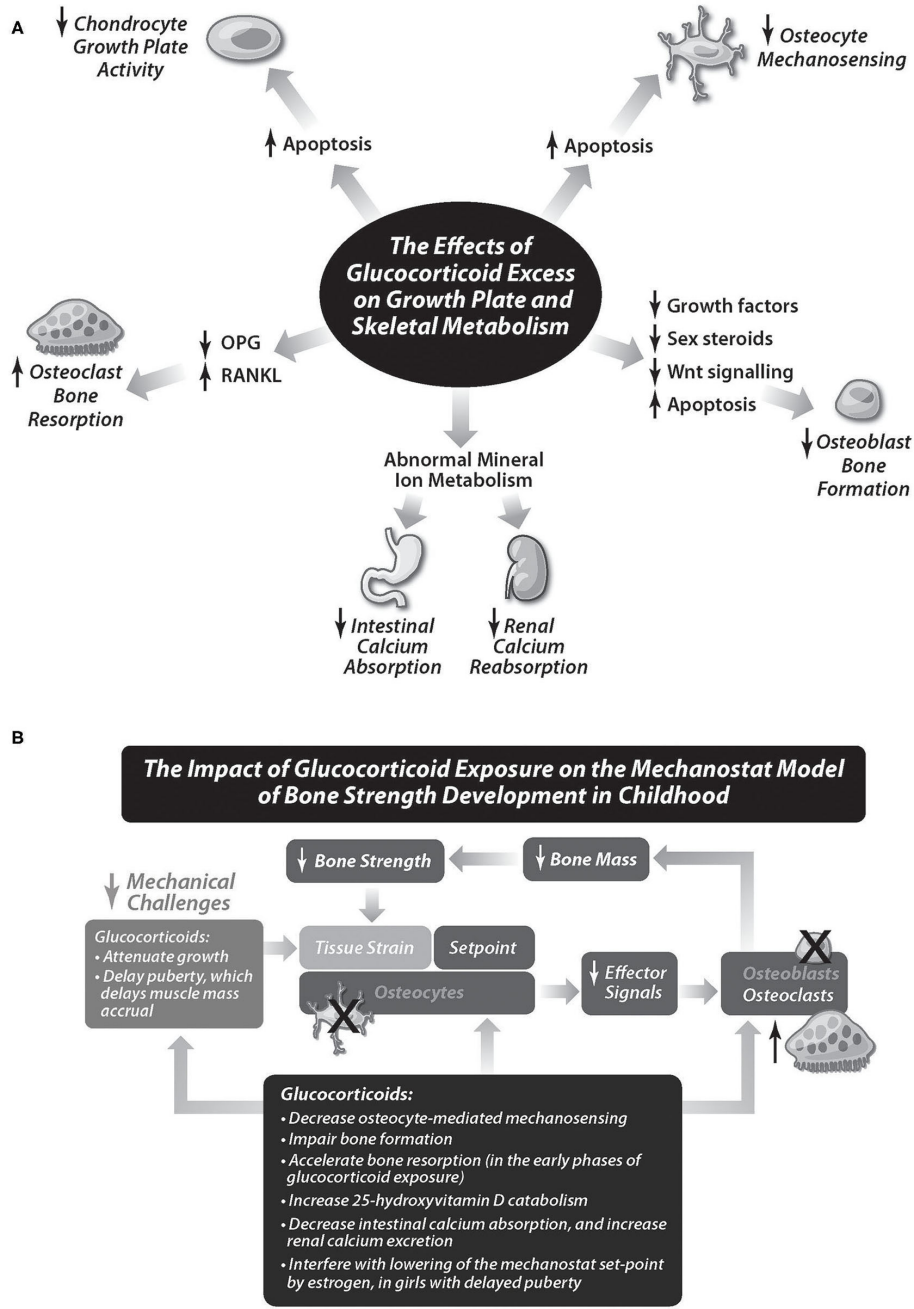
**(A)** The direct, and indirect, adverse effects of glucocorticoids on growth plate and skeletal metabolism. Together, these effects result in loss of bone strength, resulting in fragility fractures. **(B)** The impact of glucocorticoids on the mechanostat model of bone strength development in childhood. Glucocorticoids interfere with the two key mechanical challenges that normally foster bone strength in childhood—increases in bone length, and increases in muscle mass. They also have a direct, adverse effect on growth plate chondrocytes, and on all three bone cell lines (osteocytes, responsible for sensing bone tissue strain, osteoclasts, responsible for resorbing damaged bone at the site of bone issue strain, and osteoblasts, responsible for bone repair at the site of bone tissue strain, by laying down new bone). Adapted with permission from Rauch and Schoenau (9).

GC have diverse direct, and indirect, effects on the growth plate and developing skeleton, as recently reviewed in detail (7, 8), and outlined in Figure 1A. The multiplicity of adverse GC effects on skeletal strength is perhaps best understood according to the mechanostat model of bone development, as shown in Figure 1B. According to mechanostat theory, bone development is driven by two mechanical challenges during the pediatric years: increases in muscle forces, and increases in bone length (9). These two “mechanical challenges” induce bone tissue strain, which is monitored by the master bone cells—the osteocyte system. When bone tissue strain exceeds a genetically-determined threshold, osteocytes initiate effector cascades that signal osteoclasts to resorb damaged bone at the site of bone tissue strain, and osteoblasts to repair this site by laying down new bone (10, 11). These adaptive responses ensure that skeletal strength is maintained in the face of increasing mechanical challenges brought about by growth and muscle development.

Interestingly, estrogen appears to lower the threshold at which mechanical strains are sensed by the osteocyte, such that less of an osteogenic stimulus is needed to trigger the osteoclast-osteoblast response (12). The higher bone mineral content to muscle mass ratio that is observed in girls around the time of puberty is hypothesized to serve as a reservoir that can be tapped into at the time of pregnancy and lactation (9). With delayed puberty, there is loss of the estrogen-lowering effect on the mechanostat set-point in girls. Delayed puberty also leads to a reduction in muscle mass development in both sexes, which further diminishes bone tissue strain, and its subsequent positive, adaptive responses.

The adverse effect of GC on bone strength in childhood is not surprising, since GC disrupt numerous facets of the mechanostat model (7). First, GC have a profound, adverse effect on the growth plate, most often by causing chondrocyte apoptosis, and less commonly, by interfering with hypothalamic-pituitary growth hormone secretion. Attenuated linear growth, in turn, brings about loss of a potent determinant of bone tissue strain, as described earlier. Premature death of the osteocyte also appears to be a major contributor to GC osteotoxicity, resulting in loss of mechanosensing, and therefore a reduction in important effector pathways that normally coordinate adaptive changes to promote bone strength. In addition, GC cause excessive bone resorption, through promotion of osteoblast/osteocyte apoptosis, and prolongation of osteoclast survival. The bone formation pathway is also negatively impacted, since GC blunt factors which normally stimulate bone formation, including the WNT signaling system (7, 13, 14). As a result, none of the bone cells are spared the osteotoxic effects of GC therapy. It is not surprising, then, that reduced trabecular bone formation, along with increased endocortical resorption, are consistently aberrant findings that contribute to bone strength loss. The combined effects of GC on bone are not only to reduce bone mineral density (BMD) (15), but to alter bone microarchitecture, with a predilection for the trabecular-rich spine (16, 17).

**Figure 2:**
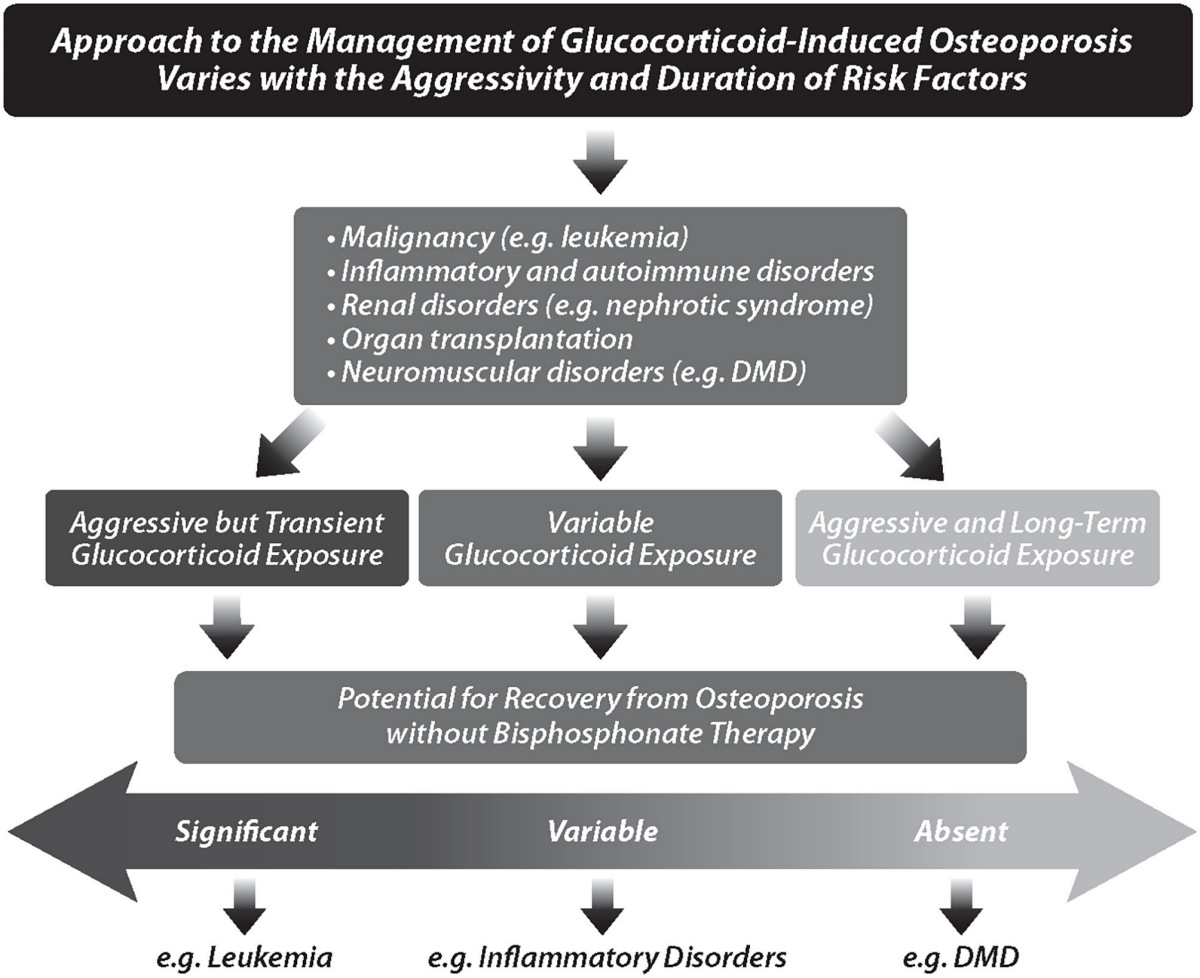
The overall approach to the management of glucocorticoid-induced osteoporosis varies with the aggressivity and duration of risk factors. Children are categorized into those with aggressive, but transient, glucocorticoid exposure, those with variable glucocorticoid exposure, and those with aggressive plus long-term glucocorticoid exposure. These categories, in turn, influence the potential for recovery from osteoporosis without bisphosphonate therapy. DMD, Duchenne muscular dystrophy.

To unravel the complexity of GC effects on the developing skeleton from a practical perspective, natural history studies have taught us to categorize GC-treated children into one of three groups (Figure 2): those with aggressive but transient GC exposure (such as children with leukemia), those with variable GC exposure (such as children with GC-treated rheumatic disorders, and nephrotic syndrome), and those with aggressive and long-term GC exposure (such as boys with GC-treated DMD). This categorization can orient the clinician to one of the most important decisions in the management of pediatric GIO—whether the child has the capacity to recover from GC-induced osteotoxicity without osteoporosis therapy. The child's ability to recover from GIO is determined by the extent to which there is sufficient residual linear growth to support skeletal modeling following transient GC exposure, vs. insufficient residual growth resulting in persistent BMD reductions, and permanent vertebral deformity following spine fractures. This “potential for medication-unassisted recovery from GIO” is pivotal to the overall approach, and will therefore be a major focus in the ensuing discussions.

## Monitoring and Diagnosis

### Clinical-Biological Principles That Inform the Early Identification of GIO, and the Decision to Treat vs. Observe

**Figure 3:**
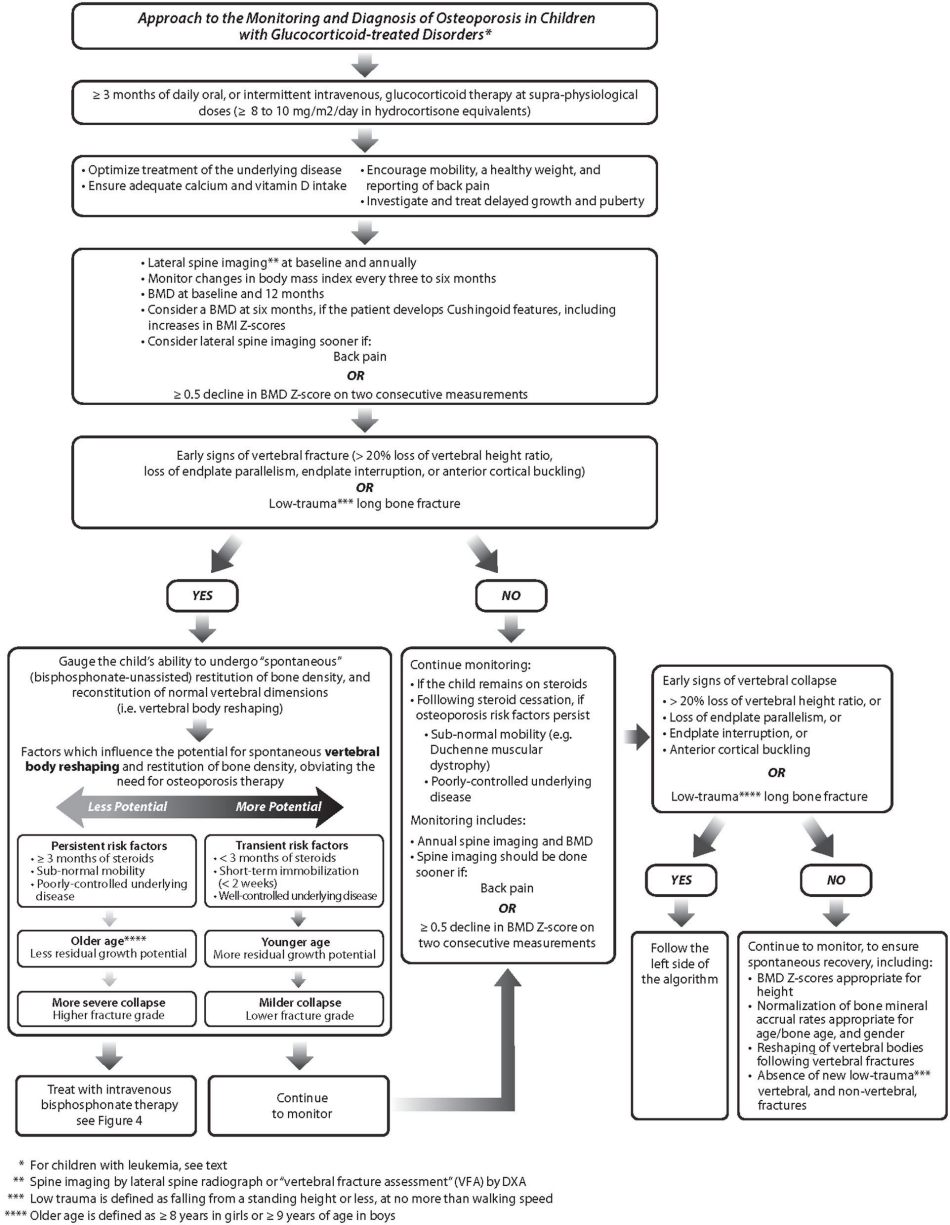
The approach to the monitoring and diagnosis of bone fragility in children with glucocorticoid-treated disorders. BMD, bone mineral density; BMI, body mass index.

**Figure 4:**
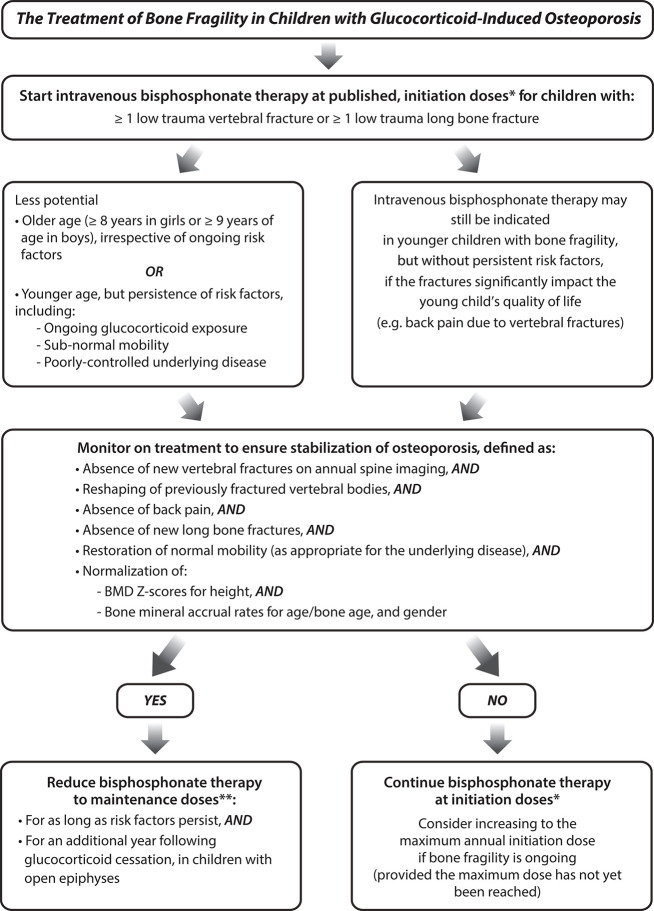
The treatment of bone fragility in children with glucocorticoid-treated illnesses. *Annual maximum initiation doses: pamidronate 4.5 to 9 mg/kg body mass/year, divided into three treatment cycles (i.e., one cycle is given every four months); zoledronic acid 0.05 to 0.1 mg/kg body mass/year, divided into two treatment cycles (i.e., one cycle is given every six months). **Annual maintenance doses: pamidronate 4.5 mg/kg/year, divided into three treatment cycles; zoledronic acid 0.025 to 0.05 mg/kg/year, divided into two treatment cycles NB: Intravenous bisphosphonate therapy for children less than 2 years of age, a rare event in pediatric GIO, is administered more frequently (i.e., pamidronate is given every two months, zoledronic acid is given every three months, same maximal annual initiation and maintenance doses as for older children).

Over the last two decades, a number of important clinical observations have informed the definition, diagnosis, and monitoring of osteoporosis in children with GC-treated diseases. Together, these observations can be distilled down to key “clinical-biological principles” representing concepts that can guide the clinician in navigating the management of any child with a GC-treated disorder. These principles are summarized in Figure 3 (monitoring and diagnosis) and Figure 4 (treatment).

#### The Diagnosis of Osteoporosis in Children Has Shifted Away From a “BMD-Centric,” to a “Fracture- and Clinical Context-Focused” Approach, With GC Exposure Representing One of the Most Important Clinical Contexts With a Higher Risk of Bone Fragility

Children with GC-treated illnesses can present with disabling complications of osteoporosis, including painful VF, permanent vertebral deformity, and premature loss of ambulation following long bone fractures in those with tenuous ambulation (such as DMD) (18–20). At the same time, fractures in the general pediatric population are frequent, with almost 50% of children experiencing at least one fracture (21, 22), and almost a quarter of children sustaining recurrent broken bones (23). In view of this, Pediatric Positions Task Forces working with the International Society for Clinical Densitometry (ISCD) over the years have sought to guide clinicians in the definition of osteoporosis in children, by developing definitions that attempt to identify children with “…an intrinsic skeletal issue resulting in bone fragility,” compared with those who break bones during play and sports (24, 25).

The most recent ISCD recommendations (24) noted that osteoporosis should not be diagnosed on the basis of solely bone density criteria; a clinically significant fracture history is also required. The ISCD definition of osteoporosis included non-traumatic VF, without the need for BMD criteria, which served to acknowledge that low-trauma VF represent an osteoporotic event in childhood. Without a VF, the ISCD definition of osteoporosis involves both a clinically significant fracture history (≥two long bone fractures by age 10 years, or ≥three long bone fractures by 19 years), and a gender- and age-matched BMD Z-score of ≤-2.0 (along with appropriate corrections for bone size). The ISCD definition also noted that a BMD Z-score >-2.0 in this context “does not preclude the possibility of skeletal fragility and increased fracture risk.”

This ISCD definition of osteoporosis in childhood (24) has been used worldwide to inform clinical practice guidelines, eligibility for pediatric osteoporosis trials, and hospital protocols. One of the successes of the definition is that it mitigates over-diagnosis of osteoporosis, and therefore unnecessary treatment of those without true skeletal fragility. This is important, because osteoporosis therapies [intravenous (IV) pamidronate, neridronate, and zoledronic acid] are not without side effects, and therefore require judicious prescription.

On the other hand, it is can be challenging to distinguish low-trauma fractures due to underlying bone fragility from fractures sustained during childhood play. When applied to the letter, the 2013 ISCD definition leads to the under-diagnosis, and thus under-treatment, of some children who would benefit from osteoporosis therapy. Why? Because waiting for a subsequent long bone fracture, or for a low BMD after a single pathological fracture, delays the start of treatment in fracture-prone children. This is a crucial point, because even a single fracture can cause permanent disability in high-risk children. Furthermore, timely initiation of osteoporosis intervention is paramount to restoring normal vertebral dimensions during the critical, rapidly-closing window of growth.

Yet another point of controversy is the inclusion of a BMD Z-score threshold in the definition of pediatric osteoporosis. It may be under-appreciated by DXA users, that age- and gender-matched BMD Z-scores produced by different DXA machines vary by as much as two standard deviations for a given child, depending on the normative data used to generate the Z-scores (26). This observation was published by three research groups using Lunar- and Hologic-derived pediatric normative data (26–28); the largest of these studies generated lumbar spine (LS) areal BMD Z-scores from all of the available pediatric reference data published in the English language, up to and including 2015 (26). Ultimately, the tremendous disparity in BMD Z-scores arising from different reference databases challenges the use of a Z-score cut-off as part of a global definition of pediatric osteoporosis. At the same time, it has been shown that the various reference databases are highly co-linear (26). As a result of the co-linearity among reference databases, the associations between LS BMD Z-scores and VF are highly similar, regardless of the normative data used to generate the BMD Z-scores (26). Therefore, the lower the BMD Z-score generated by any reference database, the more likely a child is to sustain a fragility fracture (29). A second issue that arises from the inclusion of a universal BMD Z-score threshold as part of a pediatric osteoporosis definition is that children with intrinsic skeletal fragility, including children with GC-treated disorders, can have fragility fractures at BMD Z-scores >-2.0 (18, 19, 26, 30), a fact recognized in the 2013 ISCD statement.

With these observations in mind, it has been suggested that BMD Z-scores should be viewed along a continuum that inversely correlates with bone strength, but without diagnostic cut-offs. This is because the position of the healthy BMD average, and the corresponding outer limits of normal, will vary on the continuum depending on the normative data used in a given patient to generate the BMD Z-scores (31).

An additional concern is the confounding effect of stature on DXA-based areal BMD Z-scores. The ISCD noted that appropriate adjustments should be made for short stature, and delayed puberty, when interpreting DXA-based areal BMD measurements (24). This is particularly relevant to GC-treated children, given the adverse effect of GC therapy on linear growth, and on pubertal development. The size-dependent nature of DXA-based areal BMD parameters is another reason that the fracture history figures so prominently in the diagnosis of osteoporosis among children, including those with GIO.

As a result of these issues, a more nuanced approach to the diagnosis of osteoporosis in children with GC-treated disorders factors in the child's clinical context, which includes the known risk of a fracture, the mechanism of injury (degree of trauma), and the fracture characteristics, without a specific BMD Z-score requirement (31). This approach is catalyzed not only by the limitations of BMD thresholds to define pediatric osteoporosis, but by advances in our knowledge about the natural history of osteoporotic fractures in children with GC-treated diseases, as outlined in the following sections.

#### Vertebral Fractures Are the Hallmark of GIO in the Young, but Are Frequently Asymptomatic, Necessitating Routine Spine Imaging With Validated Diagnostic Criteria

**Figure 5:**
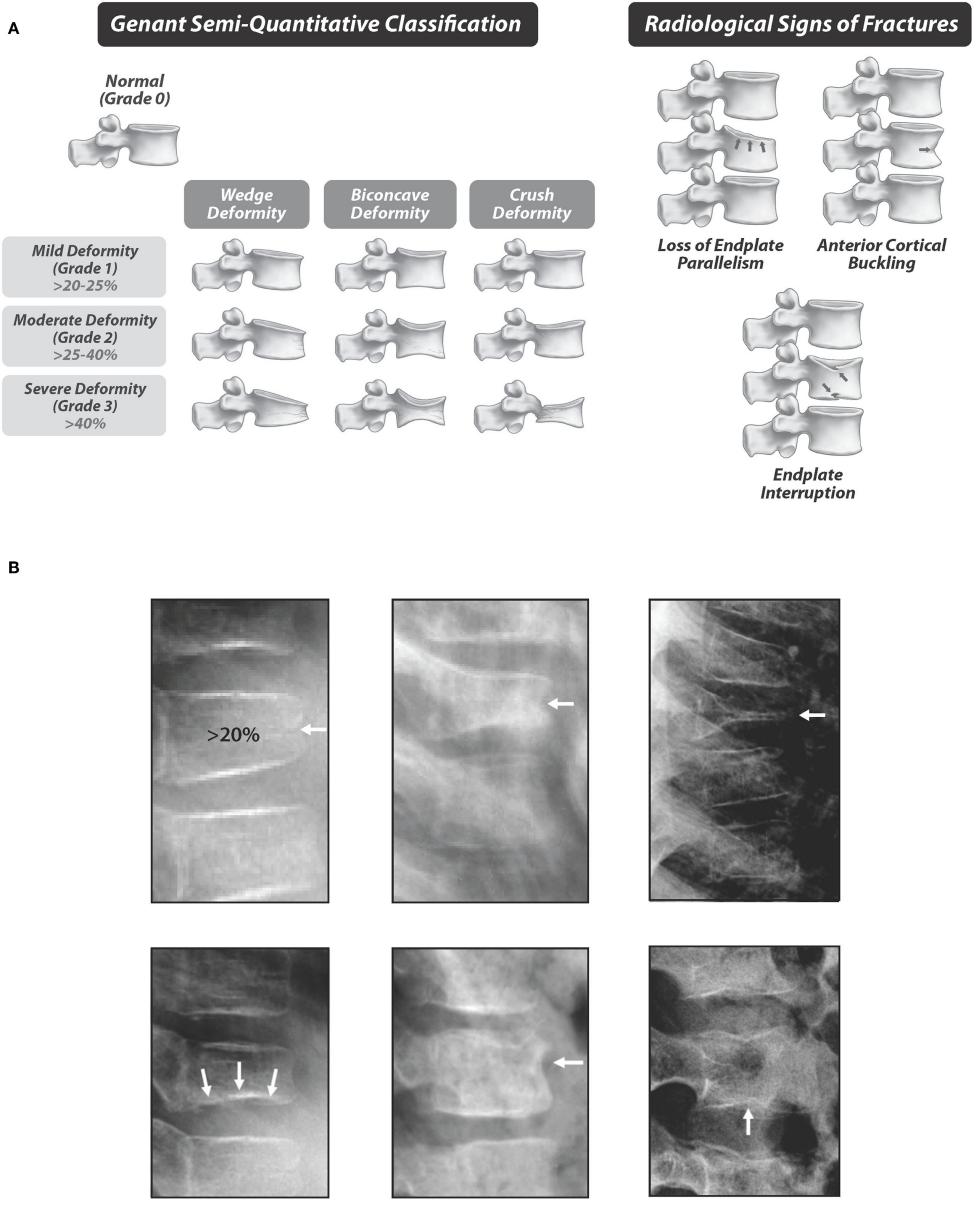
**(A)** Standardized quantification of vertebral fractures, the hallmark of glucocorticoid-induced osteoporosis in children. The depiction of the Genant semi-quantitative method is adapted with permission from Genant et al. (32). **(B)** Examples of vertebral fractures in children with glucocorticoid-treated leukemia. Top, left to right: Grade 1, 2, and 3 vertebral fractures. Bottom, left to right: Radiological signs of fractures, including loss of endplate parallelism (left), anterior cortical buckling (middle), and endplate interruption (right). Adapted with permission from Halton et al. (3).

Among the most significant findings arising from the STOPP study were that VF are the clinical signature of GIO in children, underscoring the vulnerability of the trabecular-rich spine to the adverse effects of GC. By showing that VF associate with biologically-relevant factors such as LS BMD Z-scores, back pain, second metacarpal percent cortical area, and an increased risk of future fractures (3, 19, 20, 30), the STOPP Consortium validated that >20% loss of vertebral height ratio, based on the modified Genant semi-quantitative method (32, 33), defines a VF in children (Figure 5A). The most compelling observation that validated the use of the Genant semi-quantitative method in children arose from a report in pediatric leukemia, where Genant-defined VF at diagnosis were a strong predictor of new vertebral *and* long bone fractures over the next 5 years (20). In cases where physiological anterior rounding of the vertebral body can be difficult to distinguish from a fracture, the decision can be facilitated by qualitative signs including endplate interruption, loss of endplate parallelism, and more rarely, anterior cortical buckling (Figure 5A) (34). Examples of osteoporotic vertebral fractures in children are shown in Figure 5B.

VF in children are rare in the absence of traumatic injury (21), and rates vary according to their method of detection. The highest frequencies of VF in secondary osteoporosis occur in boys with GC-treated DMD, where the VF prevalence is >50% (35), and the cumulative incidence is 28% over a median follow-up of 4 years from GC initiation (36). Children with leukemia, typically on intermittent GC therapy, have a VF prevalence of 16% at the time of diagnosis (3), and a cumulative incidence of 33% up to 6 years following diagnosis (20). In rheumatic disorders, studies have shown a 7% prevalence within 30 days of GC initiation (5), a prevalence of 29–45% later in the disease and treatment course, and up to a 33% incidence in the first few years of GC therapy, as reviewed by Hansen et al. (37).

**Figure 6:**
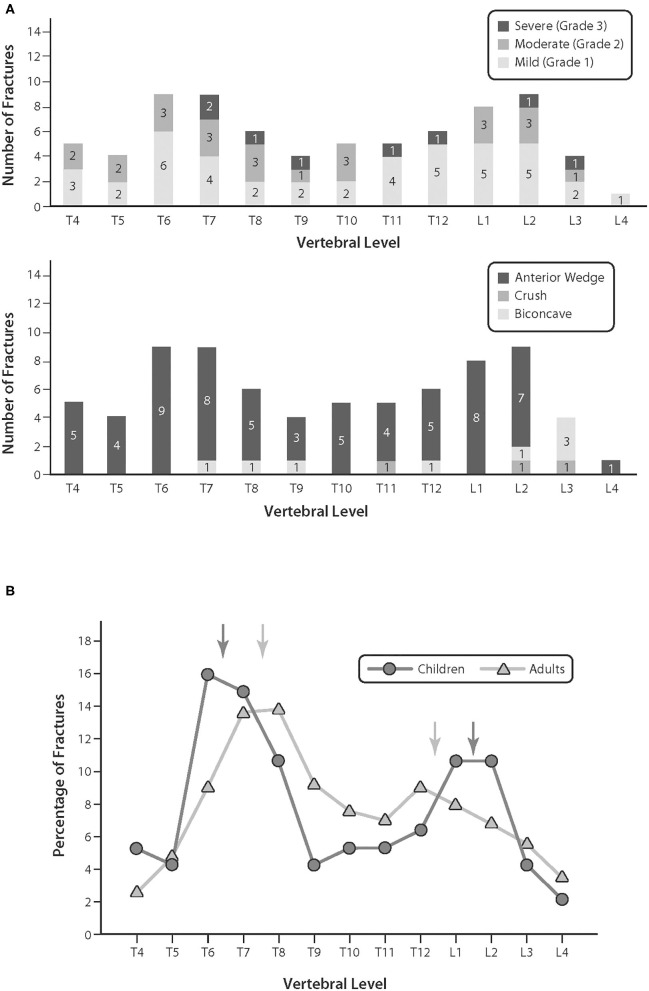
**(A)** The bi-modal distribution of vertebral fractures, and fracture morphology, in children with leukemia at diagnosis. Adapted with permission from Halton et al. (3). **(B)** The distribution of osteoporotic fractures in children compared with adults. Adapted with permission from Siminoski et al. (38).

In both children and adults, the most common VF shape is anterior wedge deformity, there is a bimodal distribution of all fracture morphologies, and the peak frequency of VF occurs in the mid-thoracic region. These are robust observations that have been demonstrated in different disease groups, at different points in the disease course (3, 18, 19, 30) (Figure 6A). The bimodal distribution of fractures in children is slightly more rostral and caudal compared with adults, as shown in Figure 6B, a finding that is attributed to the less marked thoracic kyphosis and lumbar lordosis of the immature spine (38).

VF often go undiagnosed in children with GC-treated illnesses for two main reasons. First, VF are frequently asymptomatic (3, 5, 30, 39–41), even when moderate or severe (3, 42). For example, almost half of children with VF at leukemia diagnosis were asymptomatic (3), an observation recapitulated in other pediatric GC-treated contexts (19), and well-documented in adult osteoporosis studies (43). Yet, even mild, asymptomatic VF predict future VF in children (42), an observation which underscores the importance of detecting asymptomatic disease. Secondly, surveillance with periodic spine imaging has not previously been an important part of osteoporosis monitoring in pediatric GC-treated disorders. This philosophy is changing as we shift from a BMD-centric, to a fracture-focused, diagnostic approach (31).

Given the importance of VF screening in high risk populations, there is tremendous interest in the utility of a technique called “vertebral fracture assessment” (VFA) by dual-energy x-ray absorptiometry (DXA). VFA is attractive in children, because it is an extremely low-radiation approach, which is useful when periodic VF surveillance is recommended to identify asymptomatic vertebral collapse. In addition, the fan-beam technology acquires the whole spine on a single image, obviating discrepancies in reporting due to challenges in identifying vertebral levels on two cassettes. Finally, the fan-beam technology also avoids divergence of beam issues causing parallax, making it easier to identify vertebral collapse. Newer DXA machines have a “c-arm” which rotates around the patient, obviating the need to re-position from supine to lateral when performing VFA. Image quality can vary depending on the DXA machine, as recently reviewed in detail, with newer DXA machines showing higher quality spine images (44). Recent guidelines have now been published on the use of VFA as an initial screen in children requiring periodic spine imaging for VF detection (44). Since VF detection in children involves distinguishing normal variants from pathological fractures, and since non-fracture pathology can also be seen on a VFA image, pediatric radiologists should still be involved in the assessment of VF captured by DXA.

VF have been diagnosed as early as 4 to 6 months following GC initiation in children with GC-treated rheumatic disorders and DMD (18, 30). With this in mind, bone health monitoring, including lateral thoracolumbar spine imaging, should start around the time of GC initiation in very high risk populations such as DMD (45), and as soon as possible in other diseases where children are anticipated to receive ≥3 months of daily oral, or intermittent IV, GC therapy. Lateral spine imaging should be repeated a maximum of 12 months after the initial assessment in patients who remain on GC therapy, because of the increased VF risk in the first year (30), and yearly thereafter if GC continue. Spine imaging for VF assessment is recommended sooner if there is back pain, or in the presence of ≥0.5 decline in BMD Z-score. The overall approach to monitoring is outlined in Figure 3.

#### Vertebral Fractures Can Occur Early in the GC Treatment Course, and Readily Measurable Clinical Features in the First 6 to 12 Months of GC Therapy Predict Incident VF

**Figure 7:**
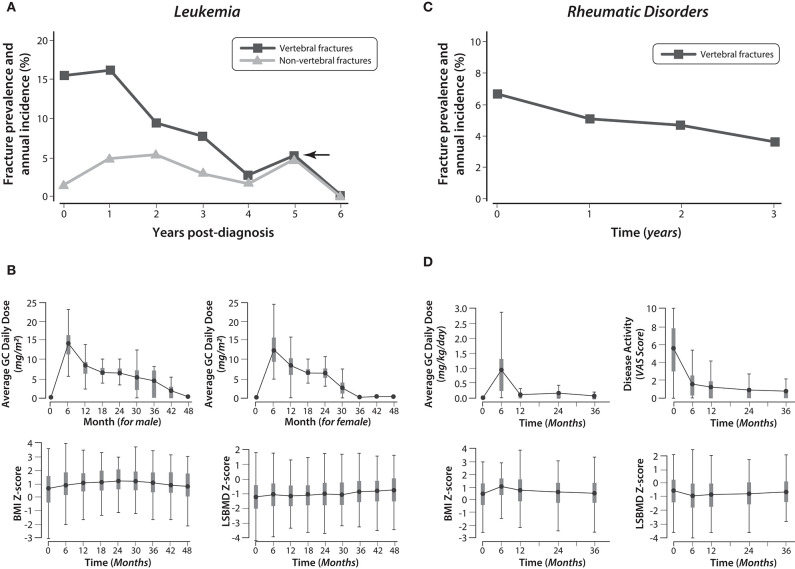
The frequency of vertebral and non-vertebral fractures from the time of glucocorticoid initiation in children with leukemia and rheumatic disorders, and the longitudinal changes in glucocorticoid exposure, body mass index, and lumbar spine bone mineral density Z-scores. Adapted with permission from LeBlanc et al. (19), Ward et al. (20), and Cummings et al. (42). **(A)** The prevalence of vertebral and non-vertebral fractures at leukemia diagnosis, and the annual incidence of fractures during the six years following diagnosis. **(B)** The changes in average GC daily dose (for boys and girls), BMI (both genders combined), and LSBMD Z-score (both genders combined) in children with leukemia during the four years following diagnosis. **(C)** The prevalence of vertebral fractures at the time of GC initiation in children with rheumatic disorders, and the annual incidence of vertebral fractures in the three years following diagnosis. **(D)** The changes in average GC daily dose, disease activity, BMI Z-score, and LSBMD Z-score in children with rheumatic disorders during the three years following diagnosis. GC, glucocorticoid; LSBMD, lumbar spine bone mineral density; BMI, body mass index.

Not only can VF occur in the first few months of GC exposure (18, 30), but the peak annual VF incidence has been shown to occur at 1 year after starting GC in both GC-treated leukemia (20), and rheumatic disorders (19) (Figure 7). This is not surprising, since the peak frequency of fractures directly mirrors the period of maximum GC exposure, along with corresponding declines in height and BMD Z-scores, increases in disease activity (for rheumatic conditions), and increases in body mass index, as shown in Figure 7 (19, 20, 30, 42).

From studies assessing the baseline and longitudinal factors associated with VF in GC-treated children, a number of useful themes have emerged. First of all, GC exposure is a consistent, independent predictor of incident VF, and both cumulative and average daily GC dose predict incident VF in a number of different diseases, as previously reviewed (46). Intermittent (pulse) therapy in children with leukemia (quantified as GC “dose intensity,” the cumulative GC dose during the observation period, divided by the number of days in receipt of GC), also predict incident VF (42).

**Figure 8:**
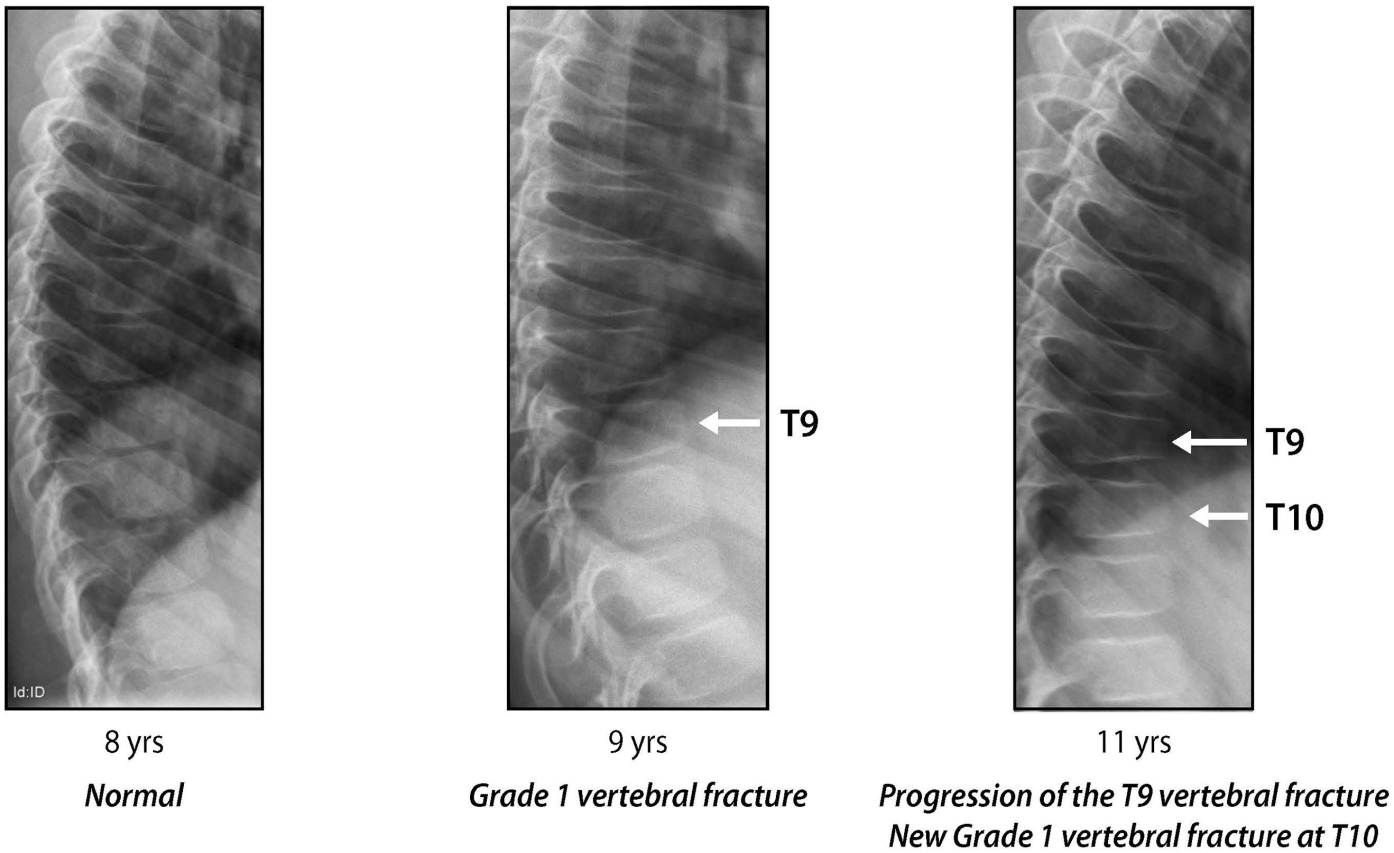
Progressive signs of vertebral collapse in a boy with glucocorticoid-treated Duchenne muscular dystrophy, in the absence of bone protection: “the vertebral fracture cascade.” Adapted with permission from Ma et al. (18).

Studies in children with GC-treated leukemia have shown that the strongest predictor of future fractures is prevalent VF around the time of GC initiation, a phenomenon known as “the VF cascade” (40, 42). Even mild, asymptomatic VF are independent predictors of future vertebral fractures, highlighting the importance of identifying early signs of vertebral collapse through periodic surveillance (40, 42). Figure 8 shows an example of the VF cascade in a boy with GC-treated DMD who has progressive VF in the absence of bone protection.

The fact that prevalent VF at the time of starting GC predict future VF underscores the importance of knowledge about the skeletal phenotype early in the child's disease course. The first year of GC therapy is also a critical time to scrutinize the child's clinical trajectory for other predictors of incident VF. In children with GC-treated rheumatic disorders, readily measurable clinical features in the first year independently predicted subsequent incident VF, including increases in body mass index in the first 6 months of GC therapy, increases in disease activity scores in the first 12 months of GC therapy, and decreases in LS BMD Z-scores in the first 6 months of GC therapy (19). In children with solid organ transplantation, older age also predicted an increased VF risk (47–50). As a general rule of thumb, any child with Cushingoid features should be considered at increased risk for VF and undergo routine spine monitoring accordingly (Figure 3). Although not tested in longitudinal studies, it is hypothesized that children with adrenal suppression due to exogenous GC therapy may also be at increased risk of VF, since adrenal hypoactivity is yet another sign of clinically significant GC exposure. While back pain was associated with VF at diagnosis in two studies, one of children with GC-treated leukemia, and the other of children with rheumatic disorders (3, 5), back pain did not predict future VF (19, 42). From these studies, we learned that the absence of back pain does not preclude incident VF in at-risk children, an observation that, in the author's experience, holds true in clinical practice.

There are no reports describing the critical GC dose or duration that is linked to an increased risk of incident VF in children, and formal prediction models to estimate absolute fracture risks in individuals do not exist. This is largely due to the small numbers of at-risk patients (relative to adults), and the fact that GC exposure is necessarily weight- or body surface area-based, with wide variability during the pediatric years. Van Staa showed a 30% increase in overall fracture risk among children with a history of four or more courses of oral GC (average duration only 5 days) (51). Studies focusing on fragility fractures specifically have reported that VF can occur around the time when GC therapy is first initiated (3, 39), that incident VF can occur as early as 4 months following GC initiation (30), and that peak annual incidences of VF mirror the period of maximum GC exposure (19, 42). These studies have informed the recommendation to screen for VF early in the child's GC treatment course, for those anticipated to remain on GC for ≥3 months (Figure 3). Since there is no evidence in children to suggest that physiological doses of GC cause overt bone fragility (i.e., ≤8 to 10 mg/body surface area in hydrocortisone equivalents), provided the underlying disease is well-controlled and itself is not a risk to skeletal health, the practical decision to initiate bone health monitoring in children is triggered by ≥3 months of daily oral, or intermittent IV, GC therapy at supraphysiological doses, as outlined in Figure 3.

#### Bone Mineral Density Remains Useful in Vertebral Fracture “Case-Finding” Paradigms, and in Tracking a Child's Bone Health Trajectory Over Time

What is the role, then, of BMD in the bone health monitoring of GC-treated children? A low BMD raises the index of suspicion for an osteoporotic fracture, but it is not diagnostic, because BMD can be low due to size artifact (i.e., short stature), and Z-scores can decline due to poor linear growth velocity, weight loss, and delayed puberty. Furthermore, BMD Z-scores can be >2.0 in GC-treated children with fractures (3, 18, 30), as previously discussed. In practical terms, BMD is but one of numerous pieces of information that orients the pediatrician as to whether the child has sustained an osteoporotic fracture, if it is not already obvious from the clinical context (i.e., a low-trauma long bone or vertebral fracture, plus a Cushingoid appearance, height deceleration, increases in body mass index, or declines in BMD Z-scores that are beyond the limits of the measurement precision).

There are two main ways in which BMD can be used to inform the clinician about the child's skeletal status in the monitoring phase. The first scenario is based on an approach which seeks to minimize radiation exposure by using clinical features to improve prevalent VF detection on spine radiographs—called “case-finding approaches.” A recent report on a large cohort of children with GC-treated acute lymphoblastic leukemia, rheumatic conditions, and nephrotic syndrome explored the accuracy of VF detection in the presence of back pain alone, low LS BMD Z-score alone, back pain *or* a low LS BMD Z-score, or a combination of back pain *and* a low LS BMD Z-score (52). Such an approach is predicated upon the known VF prevalence in the population of children in question, and acknowledges that the BMD Z-score cut-off varies according to the normative database used to generate the Z-scores. As such, the details provided in the next paragraph are specifically relevant to the cohort that was studied.

Forty-four out of 400 children with GC-treated diseases (11%) had prevalent VF in this case-finding study (52). Logistic regression analysis between LS BMD and prevalent VF gave an odds ratio of 1.9 for each reduction in Z-score unit, an area under the receiver operating characteristic curve of 0.70, and an optimal BMD Z-score threshold of −1.6. Case identification using either low BMD alone (Z-score <-1.6), or back pain alone, produced similar data for sensitivity (55% and 52%, respectively), specificity (78% and 81%, respectively), positive predictive value (24% and 25%, respectively), and negative predictive value (93% and 93%, respectively). Low BMD plus back pain showed lower sensitivity (32%), higher specificity (96%), higher positive predictive value (47%), and similar negative predictive value (92%). The approach using low BMD, or back pain, had the highest sensitivity (75%), lowest specificity (64%), lowest positive predictive value (20%), and highest negative predictive value (95%). All approaches had increased sensitivities for higher fracture grades.

With this in mind, if the clinician's focus is to minimize x-rays, a useful screening approach is the presence of a low BMD *and* back pain. This strategy would require that only 8% of this cohort would need x-rays, the approach would detect a third of patients with a prevalent VF, and it would detect an even larger number with higher fracture grades. This also means that one fracture would be found for every two patients who underwent x-rays. For a clinician who wanted to improve on this detection rate, the strategy of back pain *or* low BMD could be taken. This identified ¾ of all patients with fractures in this cohort, and had 100% sensitivity for Grade 3 VF. This paradigm provides the best strategy for ruling out a fracture, since the likelihood of a prevalent VF was only 5% in the absence of low BMD *or* back pain. On the other hand, the trade-off for the higher detection rate is that for this particular cohort, 41% would undergo x-rays, and one child would be identified with a prevalent VF for every 5 children who underwent a spine radiograph. Either way, this provides a strategy for using BMD to judiciously inform the request for x-rays in a given child, in order to detect prevalent VF. The specific strategy chosen depends on the importance of VF detection to the child's care, and the physician's attitude to radiography.

The other main utility of BMD in the monitoring phase is to signal the child with true bone loss (loss in absolute bone mineral content with declining Z-scores), or failure to gain bone at a normal rate (declining Z-scores). Declines in BMD Z-scores ≥0.4 are typically considered clinically significant based on natural history observations. For example, for every 1 g increase in cumulative GC exposure/body surface area in the first 5 weeks of GC exposure, lumbar BMD Z-scores were lower by 0.37 in GC-treated children with nephrotic syndrome (39). In a second example, this time in children with rheumatic disorders, a greater decline in LS BMD Z-score was reported in the first 6 months of GC therapy, by a difference of 0.4, in those with incident VF at 12 months compared to those without (30). While LS BMD is the site most often measured in children, recent studies have shown that total body (less head) and hip BMD are clinically sensitive in GC-treated children (53, 54). Bone mineral accrual Z-score equations were recently published; these can be explored in research studies for their ability to predict future VF (55).

#### A Single, Low-Trauma Long Bone Fracture May Represent a Major Osteoporotic Event in Those at Risk, Even Prior to GC Initiation

The overall risk of a fracture in healthy children, of which VF are exceedingly rare, ranges in boys from 42 to 64%, and in girls from 27 to 40% (22). The most frequently fractured bone is the radius/ulna, which results in nearly half of all fractures (22, 29). In addition, 65% of long bone fractures in childhood affect the upper extremities, while 7 to 28% occur in the lower extremities (22).

Since long bone fractures are extremely common in childhood, the ISCD 2013 Position Statement declared that a significant fracture history was represented by ≥2 long bone fractures by age 10 years, or ≥3 long bone fractures by age 19 years (24). These frequencies are reasonable for a child without risk factors for an underlying bone fragility condition. However, for a child with a known risk of a fragility fracture, such as those with GC-treated disorders, these criteria have been recently proposed as overly stringent (31), recognizing that other features of the fracture, and its clinical context, should be considered.

Important in the assessment of GC-treated children with long bone fractures is the definition of low-trauma. Low-trauma has been characterized in numerous ways. The 2013 ISCD Pediatric Positions Task Force defined a low-trauma fracture as one that occurred outside car accidents, or when falling from <10 feet (three meters). In GC-treated children, falling from a standing height or less at no more than walking speed has been used to define low trauma (20). This definition is valid in the chronic illness setting, because VF predicted incident low-trauma long bone fractures that were defined in this way among children with GC-treated conditions (20).

**Figure 9:**
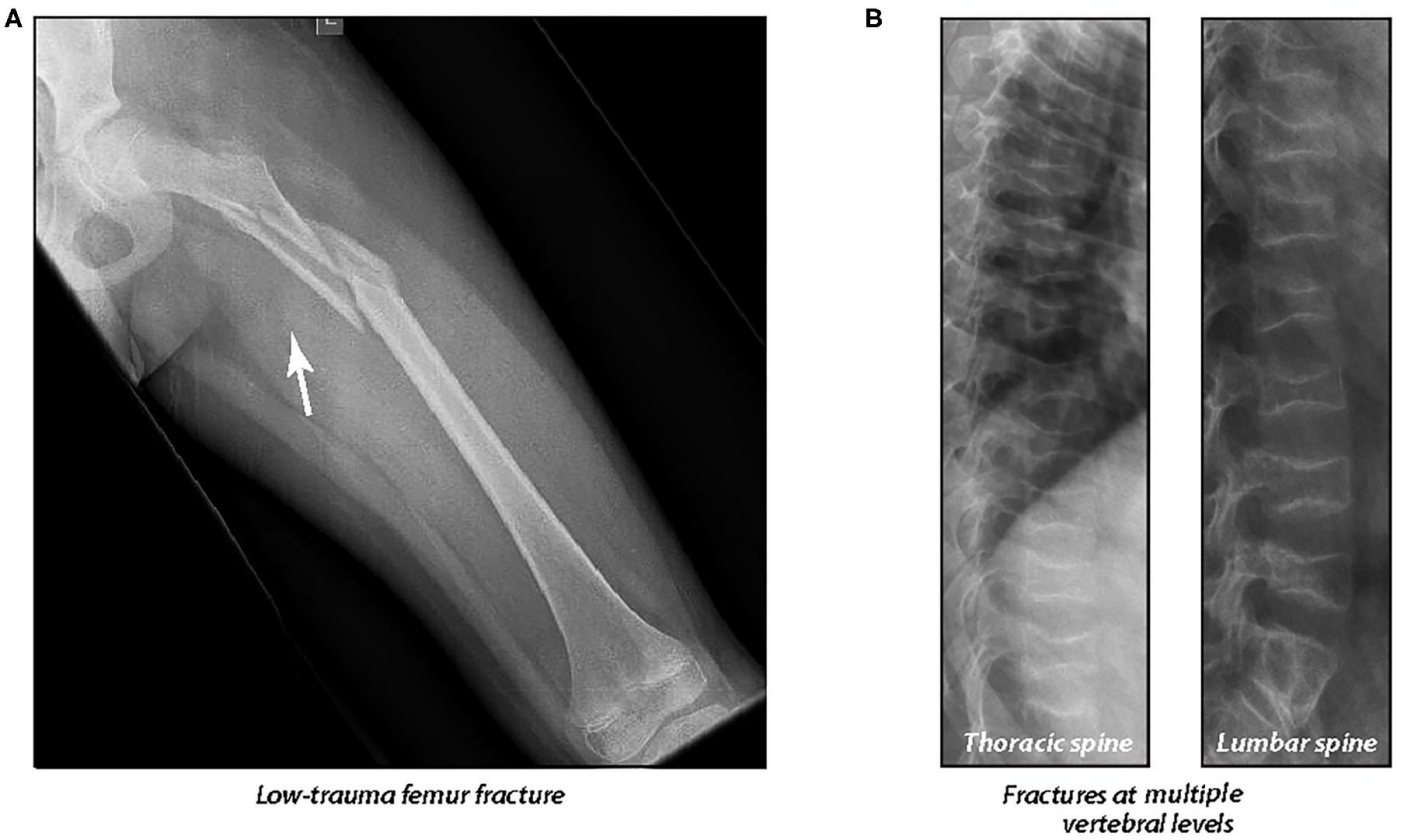
**(A)** A 10 year old boy with Duchenne muscular dystrophy who presented 2 years after glucocorticoid initiation with a low-trauma femur fracture, causing permanent, premature loss of ambulation. The low-trauma femur fracture was the patient's first osteoporotic event. Note the generalized osteopenia, gracile femoral shaft, and thin cortices. **(B)** A 13 year old boy with Duchenne muscular dystrophy who presented with multiple, painful vertebral fractures 7 years *after* a low-trauma tibia fracture. The patient's first osteoporotic event was at six years of age (at the time of the low-trauma tibia fracture). Adapted with permission from Ma et al. (18).

Lower extremity fractures are frequent in boys with DMD even in the absence of GC therapy, occurring in up to 40% (56, 57), with doubling of the long bone fracture risk in the presence of GC therapy (56). The high rate of lower extremity fractures prior to GC initiation speaks to the adverse effect of the myopathy on bone strength. In children with leukemia, long bone fractures occurred in 23% over the 5 years following diagnosis (with no new fractures between 5 and 6 years) (20). Beyond BMD, gracile bones (reduced periosteal circumference) are also characteristic of the osteoporosis phenotype in GC-naïve DMD (Figure 9A).

Even a single, low-trauma long bone fracture may be a major osteoporotic event in those with GC-treated disorders. As an example, among boys with GC-treated DMD, VF were frequent in the years *following* a single, low-trauma long bone fracture (18) (Figure 9B); this observation provided proof of principle that the long bone fracture was the child's first osteoporotic event. Lower extremity fractures typically have the greatest impact on day-to-day life because of the adverse effect on mobility. The starkest example of this arises from boys with DMD who experience premature, permanent loss of ambulation following a long bone fracture (18). This can be devastating to families living with DMD who have anticipated a certain duration of ambulation. Low-trauma femur fractures are one of the signatures of pediatric osteoporosis, but even a single tibia or upper arm fracture can represent an osteoporotic event in those at risk. Comminuted fractures, and those with atypical displacement, are also significant, especially in the absence of trauma.

Although forearm fractures are extremely common in childhood, the clinical context surrounding the fracture (low or high trauma, radiologic features), plus the GC-treated child's clinical profile (height, body mass index, puberty and BMD trajectories, GC dose and duration, presence or absence of VF, Cushingoid appearance, and disease activity) usually provides sufficient information to aid the physician in assessing the fracture's clinical significance.

#### Some Children Can Recover From GIO Through Reshaping of Fractured Vertebral Bodies and Restitution of Bone Mass, Obviating the Need for Osteoporosis Treatment

The pediatric skeleton is a dynamic structure which holds the ability to not only reclaim BMD that has been lost during transient bone health threats, but also to reshape previously fractured vertebral bodies, through a process known as skeletal modeling. Both BMD reclamation and vertebral body reshaping are important measures of recovery in children, either spontaneously or following osteoporosis therapy (i.e., bisphosphonates). Restoration of normal vertebral dimensions is thought to be growth-mediated, since it has not been unequivocally reported in adults (58).

Given the tremendous drive to recover from osteoporosis among children with transient risk factors and sufficient residual growth potential, not all children with GIO require osteoporosis intervention. Determining which children have insufficient potential for complete vertebral body reshaping following VF is a pivotal step in the management of pediatric GIO.

**Figure 10:**
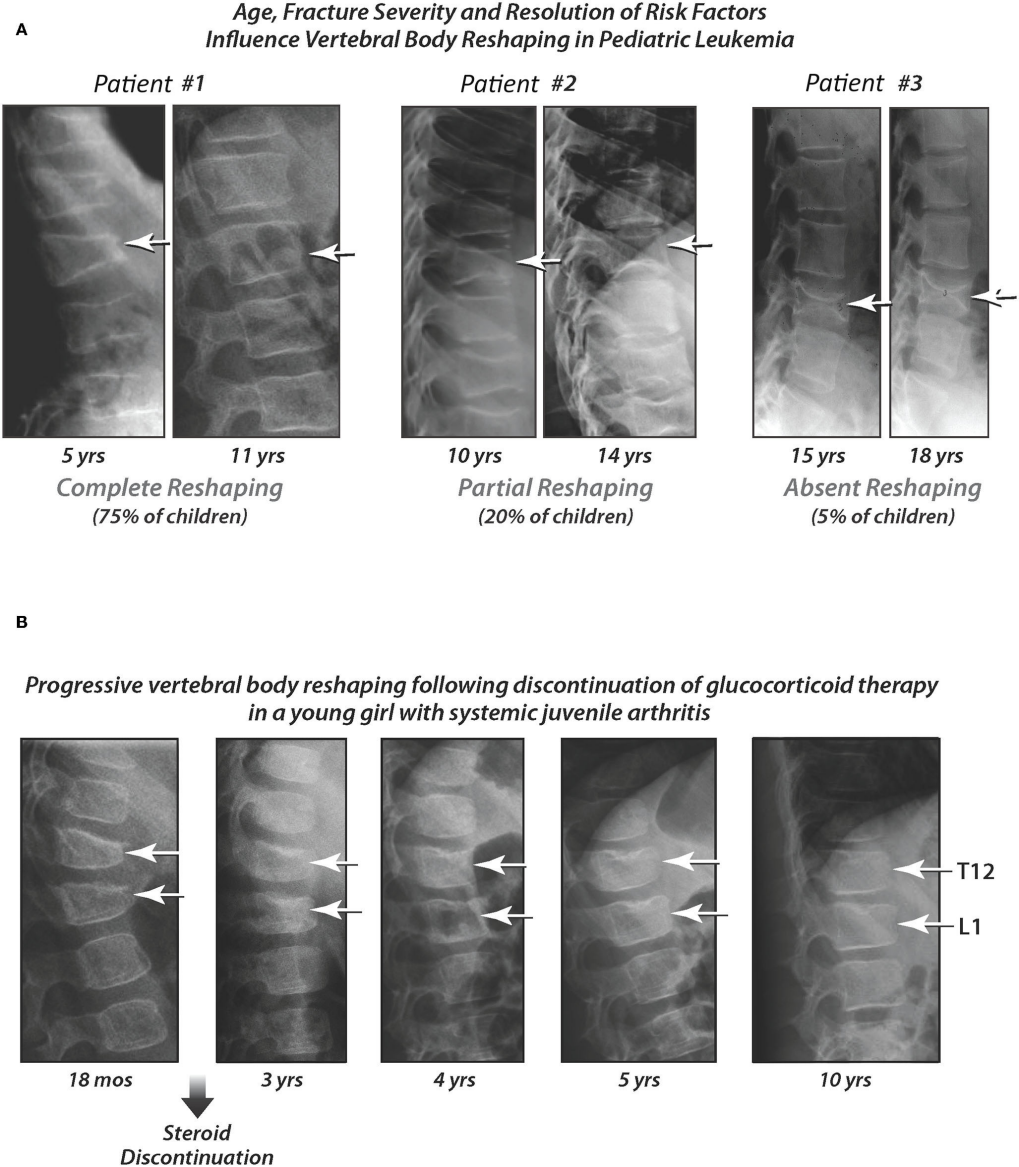
**(A)** Pediatric patients with complete, partial, and absent vertebral body reshaping following acute lymphoblastic leukemia diagnosis, in the absence of bisphosphonate therapy. The first age shown for each patient is the age at diagnosis. Patient #1 is still growing at 11 years of age, and has undergone complete reshaping. Patient #2 has finished growing at 14 years of age, and has undergone partial reshaping. Patient #3 had already reached adult height at the time of leukemia diagnosis (absent reshaping). Adapted with permission from Ward et al. (20) and Dal Osto et al. (59). **(B)** Progressive vertebral body reshaping following discontinuation of glucocorticoid therapy in a young girl with systemic juvenile arthritis. This patient developed vertebral fractures at 18 months of age (12 months after starting glucocorticoid therapy). She went on to show near-complete reshaping at 10 years of age, following discontinuation of glucocorticoid therapy, and in the absence of bisphosphonate treatment (referred to as “spontaneous vertebral body reshaping”). Adapted from Ward et al. (46) with permission.

The disease that was best-studied for signs of recovery from skeletal insult, in the absence of osteoporosis treatment, is childhood leukemia. This is unsurprising, since leukemia is a transient threat to bone health in the vast majority of children. The fact that reshaping can take place during leukemia therapy (which includes high-dose GC therapy) is hypothesized to result from the intermittent pattern of GC exposure that is the basis for current treatment protocols (Figure 10A, patient #1). Vertebral body reshaping has also been reported in children with rheumatic disorders following GC cessation (Figure 10B). Reshaping does not occur quickly, evolving over years in children with more severe collapse. Case in point, older children who lack sufficient residual growth potential can be left with permanent vertebral deformity following vertebral collapse (Figure 10A, patients #2 and 3) (20, 59). The long-term consequences of permanent deformity remain unknown; however, adult studies report reduced quality of life due to chronic back pain, and also significant functional limitations (60, 61). Whether this is true in adults who experience permanent vertebral deformity as children merits further study. In the aging, VF contribute to excess mortality (62), and among adult post-menopausal women without a history of pulmonary disease, those with VF had restrictive pulmonary dysfunction compared to those without VF (63). Together, these adult studies suggest that permanent reductions in vertebral height sustained in childhood may have important consequences later in life. The GC-treated disease where this dialogue is particularly relevant is DMD, given the shortened lifespan due to cardiorespiratory failure.

To explore the phenomenon of vertebral body reshaping further, the Canadian **STOPP** Consortium studied determinants of incomplete vs. complete reshaping in bisphosphonate-naïve pediatric patients with leukemia (20). Children who had at least one VF at any time point over 6 years following diagnosis including baseline, plus at least one spine radiograph available for VF evaluation after the first documented VF, were evaluated for vertebral body reshaping. To do this, a method known as the “spinal deformity index” (SDI) was used (64), which equals the sum of the Genant grades. For example, three Grade 1 VF is equivalent to an SDI of 3, and two Grade 3 VF equals an SDI of 6. Therefore, the SDI is a metric of overall spine fracture burden for a given child, one that can be tracked over time to quantify both incident VF, and vertebral body reshaping. In childhood leukemia, vertebral body reshaping was defined by the magnitude of the SDI decline from baseline to the last follow-up visit, as follows (20): (1) Absence of vertebral body reshaping: no change in the SDI (i.e., the last SDI was the same as the maximum SDI at previous time points); (2) Incomplete vertebral body reshaping: a decline in the SDI by <100% (i.e., 0 < last SDI < maximum SDI at previous time points); and (3) Complete vertebral body reshaping: a decline in the SDI by 100% (i.e., the last available radiograph showed the SDI was 0).

Forty-four children (23.7% of the cohort) were eligible for evaluating reshaping of vertebral bodies based on the required criteria. Using the SDI methodology, the vertebral body reshaping profile in children with leukemia was as follows (20): 77% had complete reshaping by their last follow-up visit, 18% had incomplete reshaping, and 5% had no change in the SDI. Children with incomplete or absent vertebral body reshaping were older (on average 8 years of age at diagnosis, compared with 4.8 years in those with complete reshaping), and more frequently had moderate and severe collapse at the time of the maximum SDI (90% of children with incomplete or absent vertebral body reshaping had moderate or severe vertebral collapse at the time of the maximum SDI, compared to 38.2% of children with complete reshaping). In practical terms, these data taught us that younger children, and those with less severe collapse, reshape vertebral bodies more frequently, provided risk factors for bone fragility have abated. These data further suggested that the peri-pubertal period (i.e., ≥8 years of age in girls, and ≥9 years of age in boys) was a critical point in determining whether a child had sufficient residual growth potential to effectuate vertebral body reshaping.

Because the drive to recovery among children with leukemia stands unique, the osteoporosis monitoring algorithm provided in Figure 3 does not strictly apply to this disease group. In children with leukemia, spine imaging should be conducted in any child with back pain, at any time during their leukemia treatment course, in order to identify VF. Spine imaging should also be carried out around 3 months following diagnosis, regardless of back pain, in children who are peri-pubertal or older (≥8 years in girls, ≥9 years in boys) at the time of diagnosis. The purpose of the latter recommendation is to identify older children with asymptomatic vertebral collapse outside of the induction phase, who have less residual growth potential to undergo complete vertebral body reshaping.

The next question, then, is whether children with VF, and with persistent bone health threats, can undergo spontaneous (i.e., medication-unassisted) vertebral body reshaping in the context of diseases other than leukemia. Figure 10B provides an example in a child with systemic-onset juvenile arthritis, one of the more frequently GC-treated rheumatic disorders. As evident from both Figure 10A (leukemia) and Figure 10B (an inflammatory condition), vertebral body reshaping does not occur quickly in the face of moderate to severe collapse, but rather evolves over many years. The average rate at which vertebral bodies reshape per year for a given age range has never been studied, and would be a challenging undertaking given the heterogeneity of the GC-treated diseases, and of linear growth patterns. In GC-treated DMD, where the VF frequency is particularly high, there are no published reports of vertebral body reshaping without bisphosphonate therapy. This is likely due to the long-term GC prescription, and the progressive underlying disease despite GC therapy, that is inherent to the DMD setting. As such, bisphosphonate treatment studies which demonstrate vertebral body reshaping in this context, even when uncontrolled, are showing important reversal of the progressive osteoporotic phenotype in DMD.

BMD restitution is another important index of recovery. In childhood leukemia, studies have shown degrees of BMD restitution in the years after chemotherapy has finished (65, 66). Cranial and spinal radiation predict lack of BMD restitution, particularly at doses ≥ 24 Gy (66). However, it is noteworthy that the reduction in spine areal BMD among those with radiation exposure may be due in part to growth hormone deficiency-related short stature. In leukemia survivors, other reported risk factors for incomplete BMD restitution include vitamin D deficiency, hypogonadism, and reduced physical activity (67). In practical terms, pediatric bone health clinicians look for normalization of the BMD Z-score for height as a sign of BMD restitution, and a return to a normal rate of BMD accrual for age, gender and pubertal stage. In 2019, pediatric bone mineral accrual Z-score equations were published, which may be useful in clinical practice to gauge catch-up vs. deficits in a child's BMD recovery post-insult (55). Vertebral body reshaping, normalization of BMD for height, and normalization of BMD accrual rates for age/bone and gender, are all important parts of the pediatric GIO monitoring pathways, as outlined in Figures 3, 4.

## Prevention and Treatment

### Prevention of First-Ever Osteoporotic Fractures Walks the Tightrope Between Effective Treatment of the Underlying Disease, and GC-Induced Osteotoxicity

Achieving disease remission is the cornerstone of optimizing bone strength in GC-treated disorders. The challenge, of course, is striking the balance between effective treatment of the underlying disease, and minimizing side effects including osteoporosis. This is not always possible, in which case treatment of the underlying diseases, which are often chronic, serious, and with significant adverse consequences if left poorly controlled, is the top priority. In this sense, the algorithm shown in Figure 3 seeks to safeguard against progressive, undetected osteoporosis in those where the underlying disease course necessitates ongoing GC therapy.

Concerted endeavors to minimize skeletal morbidity, while prioritizing treatment of the underlying disease, have met with variable success. In childhood leukemia, transitioning from daily dexamethasone to alternate-week dexamethasone during the delayed intensification phase led to a significant reduction in the incidence of osteonecrosis (17% vs. 9%), especially in patients ≥16 years of age (38% vs. 11%) (68). The fact that intermittent GC therapy is now the standard for many leukemia protocols is hypothesized to contribute to the high frequency of vertebral body reshaping following VF in this context (20). On the other hand, attempts to reduce GC doses in DMD, without compromising muscle function, have not been as promising. Crabtree et al. (69) studied boys with DMD on intermittent (alternating day, or weekend only) vs. daily GC therapy in relationship to anthropometry, VF and ambulatory status over 2 years. Age, and GC dose were similar at baseline. Boys on intermittent vs. daily therapy were taller (average height Z-score −0.8 vs. −1.4), lighter (body mass index Z-score +0.8 vs. +1.5), and had fewer VF after 2 years (8% vs. 40%). However, boys on intermittent therapy more frequently lost ambulation (40% vs. 20%). These data highlight that any attempts to balance effective treatment of the underlying disease against GC-induced osteotoxity need to be carried out in a disease-specific manner, with a clear understanding of the impact of different approaches on the child's overall well-being.

The idea that some GC drugs are bone-sparing has arisen from studies of prednisone, methylprednisone, and deflazacort, specifically in children following renal transplant, and in children with chronic arthritis. However, comparisons among different GC preparations were made challenging in these studies by the fact that the calculated steroid dose equivalencies were heterogeneous. Disease outcomes were positive in the deflazacort-treated patients, including improvements in anthropometry, and in BMD parameters (70–72). On the other hand, a recent publication raises doubt about the bone-sparing nature of deflazacort, since bone fragility is frequent in deflazacort-treated boys with DMD (35). This observation is undoubtedly, at least in part, related to the high, long-term doses used in DMD.

In view of the side effects of traditional GC therapy in pediatric DMD, where GC are prescribed in the spirit of long-term and high-dose use, international efforts are currently underway to understand the relative benefits, and risks, of different GC regimens that are currently used in routine clinical care. A large multi-national, double-blind, randomized controlled study studying the three more commonly-prescribed GC regimens, called “**F**inding the **O**ptimum **R**egimen for **D**uchenne **M**uscular **D**ystrophy” study (**FOR-DMD**, ClinicalTrials.gov Identifier NCT01603407) is currently underway to study prednisone 0.75 mg/kg/day, prednisone 0.75 mg/kg 10 days on, 10 days off (intermittent therapy), and deflazacort 0.9 mg/kg/day (73). The results of this ground-breaking study are highly anticipated.

The narrow therapeutic window of traditional GC therapy raises the need for an effective therapy that holds fewer systemic side effects than traditional GC such as prednisone and deflazacort. Vamorolone (VBP15) is a “dissociative steroid” that holds such promise (74)—dissociative because it retains the beneficial trans-repression (anti-inflammatory) activity of traditional GC but with a significant reduction in trans-activation (hormonal gene transcription) effects (75). Phase I studies have been carried out in healthy adults and confirm a lack of short-term adverse effects of vamorolone on bone turnover markers compared to prednisone (76). Phase IIa pharmacokinetic and dose-finding studies in pediatric DMD have also been successfully completed with favorable results, as recently published following 24 weeks of vamorolone therapy (77). Longer-term and placebo-controlled studies are presently underway.

Other prevention measures include timely identification and treatment of endocrinopathies, encouraging mobility within the limits of the underlying disease, and treating nutritional deficiencies. Delayed puberty is a frequent consequence of GC therapy, as are delays that arise from poorly-controlled disease. In boys with GC-treated DMD, hypogonadotrophic hypogonadism is frequent, and recent care considerations have encouraged treatment with testosterone for those with delayed puberty (45, 78).

Poor growth is also a feature of GC therapy, most often due to a direct adverse effect of GC on the growth plate, rather than abnormal GH secretory status. In patients with recombinant human growth hormone (rhGH)-treated inflammatory disorders, the effect of rhGH on height has been modest at best, with most trials nevertheless describing a favorable effect on muscle and bone (79, 80). However, some children also experienced adverse events, including glucose intolerance, reactivation of the underlying disease, and osteonecrosis (79, 80); whether these side effects were related to the rhGH, to concomitant GC administration, or to other factors, is unclear.

Short stature is a significant problem for boys with GC-treated DMD (78), particularly during the ambulatory phase when height differences are most noticeable. The main effect of rhGH on bone strength is via improved muscle strength; however, muscle damage and fibrosis begin early in the life of a boy with DMD. Therefore, it is unlikely that rhGH would be a major modifier of bone strength in this context. Furthermore, whether rhGH might cause muscle damage in DMD over the longer term is unknown.

The effect of rhGH on height in pediatric DMD was tested in an uncontrolled pre-post study (average age 11.5 years, 39 boys), which showed an increase in height velocity, on average, from 1.2 cm in the year before rhGH therapy, to 5.3 cm in the year while on rhGH (81). The therapy appeared to prevent a decline in growth velocity, with height Z-scores stabilizing on average at −2.9 (81); however, the impact of rhGH on skeletal maturation was not measured, rendering the potential impact on adult height uncertain. rhGH did not impact the velocity of muscle or cardiopulmonary decline in this short-term study. Three patients experienced side effects (benign intra-cranial hypertension, worsening of scoliosis, and impaired fasting glucose). Given the cost of rhGH, the burden of sub-cutaneous injections that are given multiple times per week, the potential for important adverse events, and questions about long-term safety, the benefits of rhGH to prevent osteoporosis in DMD, outside of hormone replacement for those with a truly deficient secretory status, do not seem to justify the risks, costs, and inconvenience at the present time.

Non-pharmacotherapeutic measures to optimize bone health, including weight-bearing physical activity, nutrition, and maintenance of a healthy weight are also important, with excessive weight gain being a major complication in GC-treated children undergoing therapy at supra-physiological doses (78, 82). Given the complexity of care involved in the management of a child with GIO, a multi-disciplinary team is typically implicated, including the physician most responsible for the treatment of the underlying disease, the bone health clinician (often an endocrinologist given the links to growth, puberty, calcium, and vitamin D metabolism), an orthopedic surgeon, a radiologist for vertebral fracture ascertainment, a bone densitometrist with experience in acquiring DXA scans in children, and allied health professionals for the purpose of physiotherapy/physical activity prescription, psychological support, and nutritional counseling.

The most well-described nutritional factors to foster bone strength are vitamin D and calcium; however, numerous other nutrients also influence skeletal strength, including vitamins A, C, and K, iron, copper, fluoride, zinc, protein, potassium, and magnesium.

GC decrease synthesis, and increase catabolism, of vitamin D, putting GC-treated children at increased risk for vitamin D deficiency even beyond classic factors such as Northern latitudes, darker skin color, obesity, and low vitamin D dietary intake (83). The recommended intake of vitamin D is at least 600 IU/day (84), though higher doses may be needed to meet target levels, particularly in children with multiple risk factors for vitamin D deficiency. Vitamin D adequacy has been defined at a serum 25-hydroxyvitamin D (25-OHD) threshold ≥50 nmol/L (20 ng/mL) (84, 85) or ≥75 nmol/L (30 ng/mL) (86), largely based on adult studies. In children, the optimal serum 25-OHD threshold remains controversial. A meta-analysis did not show a significant effect of vitamin D supplementation, or 25-OHD levels ≥50 nmol/L, on BMD in healthy children (87). Similarly, calcium plus vitamin D supplementation had no effect on spine BMD in children with inflammatory bowel disease (88), nor in children with leukemia (89). From a practical perspective, a 25-OHD level ≥50 nmol/L (20 ng/mL) is recommended through diet and/or supplementation combined, with measurement of 25-OHD levels in GC-treated children annually, ideally at the end of winter to determine the 25-OHD trough.

The Institute of Medicine (84) has established age-specific dietary reference intakes for calcium across the lifespan. The recommended dietary allowance of calcium to fulfill the needs of 97.5% of the healthy pediatric population is 700 mg/day for children 1 to 3 years of age, 1,000 mg/day between 4 and 8 years, and 1,300 mg/day for those 9 to 18 years of age (84). Higher daily supplementation may be required in children on GC therapy. Optimizing calcium intake through diet is preferred, because of questions raised following reports of adverse cardiovascular outcomes in adults on supplementation (90), and due to concerns about exacerbation of hypercalciuria in children with GC-treated mobility disorders.

### Calcium, Vitamins D and K, Vitamin D Analogs, and Oral Bisphosphonate Therapy Are Relatively Weak Modulators of BMD in GC-Treated Children, and Do Not Appear Effective in Preventing Fragility Fractures

The role of calcium, vitamin D, vitamin K, and vitamin D analog therapy for the prevention of pediatric GC-treated diseases has recently been reviewed by Jayasena in a small number of studies with few patients (91). These studies showed at best modest LS BMD improvements, or prevention of decline, in treated patients compared with controls (92–94).

Since BMD is often low at the time of GC initiation in children with serious underlying diseases (3, 5, 95), the ideal preventative therapy would not only mitigate declines in BMD, but reverse the BMD downward trajectory. The role of alfacalcidol in the optimization of BMD was studied in a recent large, randomized, controlled trial of GC-treated children (*n* = 217) with rheumatic disorders, and showed that LS BMD Z-scores declined similarly after 1 year in both the alfacalcidol and placebo groups (96). The same study further showed that the difference in LS BMD Z-score change over 1 year on risedronate compared with placebo was 0.274 (*p* < 0.001), and on risedronate compared with alfacalcidol was 0.326 (*p* < 0.001), both favoring the risedronate group. Although the trial was not powered to assess differences in fracture rates, it is nevertheless concerning that in this study (96), three children on risedronate had incident VF at 12 months, including one child who progressed from absence of fractures at baseline, to a severe (Genant Grade 3) VF at 12 months. Even more concerning was that bone resorption and formation markers increased on risedronate (96), when a decline in bone turnover markers is the biochemical signature of effective anti-resorptive therapy (97).

In a non-randomized trial of oral risedronate vs. no treatment in pediatric GC-treated DMD, 5/15 patients in the untreated group had incident VF, compared to 3/52 in the risedronate treated arm. While encouraging, the controls were also treated with GC on average 1.4 years longer than the risedronate-treated group, and the duration of GC therapy was a significant negative predictor of the change in LS volumetric BMD Z-score. Furthermore, there was no difference in the change in LS volumetric BMD Z-score, nor in the change in total body bone mineral content (less head) Z-score, over the 3.6 years of observation.

Together, these studies do not provide compelling evidence to prescribe nutritional supplements for the prevention of GC-related declines in BMD, beyond general bone health measures such as ensuring recommended intakes of calcium and vitamin D. Furthermore, oral risedronate given preventatively appears to increase BMD compared to no treatment, and compared to alfacalcidol, in pediatric GC-treated rheumatic disorders, but does not bring about the expected decline in serum bone resorption markers, and does not appear to prevent significant VF progression. These conclusions are similar to those arising from the osteogenesis imperfecta literature (98).

### The Ideal Candidates for Osteoporosis Treatment Are Children With Early (Rather Than Late) Signs of Bone Fragility, Plus Lack of Potential for Spontaneous Recovery

If prevention fails, and a child sustains a low-trauma vertebral or long bone fracture, the next step is to gauge the child's capacity to undergo “medication-unassisted” recovery from osteoporosis. Children who are younger, with transient GC exposure and sufficient residual growth potential, are more likely to recover, and can be monitored optimistically provided they are not suffering from undue back pain. Indeed, significant back pain from VF is an absolute indication for bisphosphonate therapy, irrespective of the child's capacity for spontaneous recovery from GIO. On the other hand, older children (girls ≥ 8 years, and boys ≥ 9 years) with less residual growth potential, and those with ongoing bone health threats regardless of age, have less capacity for spontaneous reshaping of vertebral bodies, as described in an earlier section. Understanding the child's clinical trajectory and ongoing GC needs is an important part of the “potential for spontaneous recovery assessment,” one that often includes speaking to the child's attending physician in order to understand the projected plan around GC prescription.

Overall, the goal of monitoring high-risk children is to identify early- rather than late-stage osteoporosis, in order to activate strategies that prevent progression (i.e., secondary prevention). The other aim of monitoring for early osteoporosis identification is to capitalize on the synergistic effects of anti-resorptive therapy during growth. Because VF are a key manifestation of pediatric GIO, one of the over-arching aims is to avoid leaving a child with permanent vertebral deformity at the time of epiphyseal fusion. These concepts provide the impetus for the approaches outlined in Figures 3, 4.

### Intravenous, Not Oral, Bisphosphonates Are the Recommended First-Line Therapy for Treatment of Pediatric GIO

Although IV bisphosphonates are the most frequently prescribed agents for pediatric bone fragility, regardless of etiology (46, 99, 100), they remain off-label worldwide with the exception of neridronate in osteogenesis imperfecta (Italy). Most data on IV bisphosphonates have arisen from the pediatric osteogenesis imperfecta literature, where it has been shown that children receiving IV pamidronate, neridronate, and more recently zoledronic acid, demonstrate improvements in lumbar BMD Z-scores, vertebral height ratios, muscle strength, activities of daily living, cortical thickness on trans-iliac biopsies, and reductions in long bone fracture rates (101–103). The evidence to support the recommendation that oral bisphosphonates should not be used as first-line therapy in pediatric GIO is provided in the section entitled: *Calcium, vitamins D and K, vitamin D analogs, and oral bisphosphonates are relatively weak modulators of BMD in GC-treated children, and do not appear effective in preventing fragility fractures*.

In pediatric GIO, placebo-controlled trials are ideal, given observations of medication-unassisted recovery when GC exposure is transient, and the frequency of disease-related symptoms that can mimic the first-infusion side effects of IV bisphosphonate therapy. However, in contrast to the osteogenesis imperfecta literature, there have been relatively few studies on the response to IV bisphosphonate therapy in pediatric GIO, in part due to challenges studying a population with heterogeneous underlying diseases and treatments, a lower frequency of fractures compared to osteogenesis imperfecta, and unpredictable relapses and remissions. In pediatric GIO, the only randomized, controlled trial of IV pamidronate, compared with oral calcium and calcitriol, was prematurely abandoned over 15 years ago (104). With a target sample size of 30 in each group in order to achieve sufficient power on the change in lumbar BMD Z-score, this study enrolled a total of 12 patients over 4 years before halting the trial due to irreconcilable feasibility issues (104).

On this background, it is not surprising that there have been only two other controlled trials studying the efficacy and safety of IV bisphosphonates specifically in children with GIO; these studies were non-randomized, case-control trials conducted over a decade ago, each on small numbers of patients (105, 106). The first study examined IV pamidronate in 17 GC-treated children with fractures who had underlying renal and rheumatic disorders, compared to an equal number of treatment-naïve controls matched for age, gender, disease and GC exposure (106). The second report assessed IV alendronate in five GC-treated children with rheumatic disorders, compared to six untreated controls for whom the clinical characteristics were not described (105). Obviously, neither study was powered to assess differences in fracture rates over 1 or 2 years of observation; however, between-group increases from baseline were significant at the spine on monthly IV pamidronate compared to the control group (106), and a transient flu-like illness was reported in 18% of patients following the first dose. On the other hand, no side effects were observed in the smaller study of five children who received IV alendronate every 3 months. In this alendronate study, within-group changes from baseline were positive at the femoral neck compared with lack of change from baseline in the untreated controls (105).

While IV pamidronate has historically been the most frequently used bisphosphonate (46, 99), in recent years, zoledronic acid has been of interest due to the numerous indications for its use in adults, a shorter infusion time, and a longer duration of action compared with pamidronate. IV zoledronic acid is the most potent bisphosphonate available, approved globally to treat osteoporosis in men, in post-menopausal women, in adult GIO, and in the prevention of future fractures in adults with a prior history of low-trauma hip fractures.

Two small, uncontrolled studies on the use of zoledronic acid in childhood osteoporosis, including GC-treated illnesses, showed improvement in BMD and absence of new VF (107, 108). Further, IV pamidronate or zoledronic acid given for 2 years in a retrospective observational study of boys with GC-treated DMD who had a total of 27 painful VF (67% percent of which were moderate or severe VF), showed improvements in back pain, stabilization or improvements in vertebral height ratios of previously fractured vertebral bodies, and an absence of incident VF (109). The stabilization or reshaping of VF on IV zoledronic acid or pamidronate, albeit on a small number of patients with high fracture burden, was nevertheless an important observation in GC-treated DMD, because medication-unassisted vertebral body reshaping following VF has never been described. This is not surprising, given the high doses of GC used to treat this condition, and the relentlessly progressive myopathy. Finally, a randomized trial of zoledronic acid (*N* = 7) versus IV placebo (*N* = 6) in children with Crohn's disease (two of whom had received GC therapy in each group) showed a greater increase in LS BMD Z-score at 6 months on zoledronic acid (+0.7) versus placebo (+0.1, *p* < 0.001). A 50% decline in urinary C-telopeptide of type I collagen was also observed, compared with no change on placebo (110).

One of the questions that is frequently asked is whether oral bisphosphonates can supplant IV therapy for the treatment of pediatric osteoporosis, including GIO. This is an attractive option, given their convenient route of administration and fewer side effects. However, the bioavailability of oral bisphosphonates is low (111). Furthermore, evidence from randomized, placebo-controlled trials of oral bisphosphonates in children with osteogenesis imperfecta does not support the use of oral instead of IV agents, as reviewed extensively elsewhere (46, 98). The recent randomized controlled trial of oral risedronate in pediatric GC-treated rheumatic disorders described earlier under prevention of GIO, also fails to provide sufficient evidence to support the use of oral bisphosphonates for the treatment of pediatric GIO (96).

Another question that frequently arises in the management of pediatric osteoporosis, is whether bisphosphonate therapy should be interrupted following a long bone fracture. To date, there has been no evidence in children with GIO, nor in children with primary bone fragility such as osteogenesis imperfecta, that healing is delayed following spontaneous fractures in children on intravenous bisphosphonate therapy. Therefore, based on present knowledge, it seems reasonable to continue bisphosphonate therapy after a long bone fracture in pediatric GIO.

The side effects of IV bisphosphonate therapy are not trivial, especially in children with underlying diseases (99, 112). The most frequent is “the acute phase reaction,” most marked following the first dose, consisting of fever, myalgias, arthralgias, bone pain, nausea, and vomiting. The acute phase reaction can precipitate signs and symptoms of adrenal insufficiency in those with adrenal suppression, necessitating “steroid stress dosing.” Hypocalcemia is also frequent in the first few days following IV bisphosphonate therapy. Although typically mild and asymptomatic, hospitalization for an IV calcium infusion has been rarely described (99, 112). The acute phase reaction is typically self-limited to a few days, aided by supportive care to minimize the intensity and duration including anti-pyretics, anti-inflammatories, and anti-nauseants. Pre- and post-treatment vitamin D and calcium supplementation are also recommended. Osteonecrosis of the jaw, a concern highlighted in the adult bisphosphonate literature, has not emerged as an issue in children despite almost 30 years of IV bisphosphonate prescription. In addition, there are no reports of atypical femur fractures in pediatric GC-treated conditions. Given the potency of IV bisphosphonate agents, combined with the potential for side effects that appear to be amplified in children with secondary osteoporosis, their prescription by a physician experienced in their use is recommended. Additional discussion on the side effects of IV bisphosphonate therapy and their management is provided elsewhere (46).

**Figure 11:**
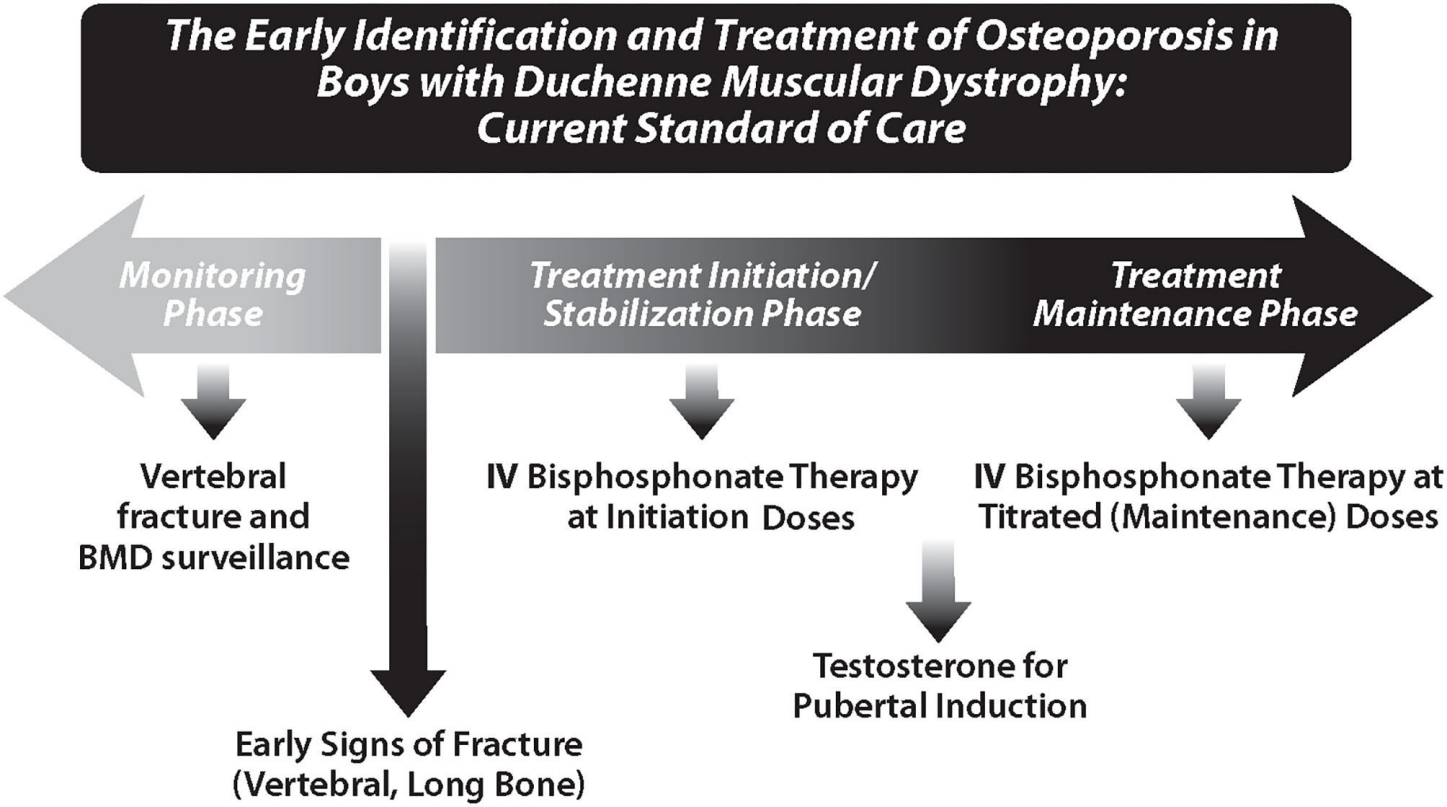
The diagnosis and treatment of osteoporosis, and delayed puberty, in boys with Duchenne muscular dystrophy. This is an internationally-endorsed, osteoporosis identification and treatment paradigm in a patient population with long-term glucocorticoid prescription [as described in Birnkrant et al. (45)].

Taken together, the current recommendations for the treatment of pediatric GIO are similar to other osteoporosis conditions of childhood, and involves the initiation of IV bisphosphonate therapy at early, rather than late, signs of bone fragility *in children with limited potential for spontaneous recovery*. These principles are reiterated in Figures 3, 4. Figure 11 provides an example of these recommendations, summarily compiled in internationally-endorsed clinical care considerations for patients with GC-treated DMD (45, 78). IV bisphosphonates should be prescribed to children by physicians who are experienced in their use, and signs and symptoms of adrenal insufficiency should be promptly identified and treated if they arise following the first infusion.

In patients with aggressive forms of GIO such as DMD, both published and unpublished experience teaches that IV bisphosphonate therapy may not rescue the phenotype in all cases. In the study of VF outcomes on IV bisphosphonate therapy over 2 years by Sbrocchi et al. (109), there were two boys who had loss of vertebral height in one vertebral body each. Neither vertebral height loss reached fracture grade (i.e., the degree of vertebral height loss was <20%); however, the reduction in vertebral height ratio, albeit minimal, was nevertheless clear with longitudinal observation. This finding is hypothesized to be influenced by the extent of vertebral collapse at the time of IV bisphosphonate initiation, with greater collapse conferring a lower threshold for triggering future VF, known as “the vertebral fracture cascade.” The magnitude of the ongoing risk factors is likely also a contributing factor to progressive vertebral height ratio loss. These observations provide rationale for initiating intravenous bisphosphonate therapy prior to the first-ever fracture. To date, there are no published studies of intravenous bisphosphonate treatment which have tackled prevention of first-ever fractures in a high risk population such as DMD.

### The Synergistic of Effects of Anti-resorptive Therapy Combined With Linear Growth Provide the Rationale for Not Withholding Bisphosphonate Therapy From a Child With Low Bone Turnover Osteoporosis

Another question that is frequently asked in pediatric GIO, and in other osteoporotic conditions of childhood characterized by low bone turnover, is whether the prescription of an anti-resorptive agent is prudent. Indeed, low bone turnover is the hallmark of pediatric GIO, which has been verified on trabecular surfaces through trans-iliac bone biopsies (113). While the use of an anti-resorptive is notionally imperfect in low bone turnover states, it is important to recognize that withholding bisphosphonate therapy in low bone turnover *prior to* growth cessation will prevent positive, growth-mediated skeletal effects arising from the unique synergy between anti-resorptives and bone modeling, as outlined in the following paragraph.

At the level of the vertebral body growth plates, bisphosphonates do not interfere with endochondral bone formation (the bone modeling process which increases vertebral height), since bone turnover on trabecular surfaces, which is suppressed by bisphosphonate therapy, is unlinked to endochondral bone formation. As a result, fractured vertebral bodies can reshape by endochondral bone formation *despite* low trabecular bone turnover, *provided a child has some degree of residual linear growth*. Bisphosphonates thereby have a permissive effect on vertebral body reshaping by increasing BMD, thereby allowing growth-related bone modeling to take place in an unfettered fashion.

This principle has been nicely demonstrated in boys with GC-treated DMD. On trans-iliac bone biopsies, further declines in the already-low bone formation at trabecular surfaces pre- vs. post-IV pamidronate and zoledronic acid nevertheless were associated with increases in vertebral dimensions through endochondral bone formation (109). The caveat here is that the extent of vertebral body reshaping is directly related to linear growth potential, and whether the insult to bone (such as GC exposure) is ongoing, attenuated, or withdrawn. To this end, reshaping of previously fractured vertebral bodies is not a realistic goal for bisphosphonate-treated children with ongoing GC exposure *and* poor linear growth. In such cases, the goals of therapy at the level of the spine are limited to prevention of incident low-trauma VF, and minimization of back pain.

Along the same lines, periosteal apposition is the growth-dependent process by which long bones increase in width as they growth in length, in order to maintain bone strength close to a genetically-determined set-point. Bisphosphonates decrease resorption on endocortical surfaces; however, periosteal apposition continues on exocortical surfaces, giving rise to a net increase in cortical thickness during growth (114). This is because bone resorption (on endocortical surfaces), and growth-mediated bone formation (on exocortical surfaces), are uncoupled at the cortex (in contrast to the trabeculae where the two processes are linked). This causes a net increase in long bone cortical width during bisphosphonate treatment while patients are growing, a phenomenon first demonstrated by Rauch et al. in children with osteogenesis imperfecta (114). Just like vertebral body reshaping, poor linear growth will also attenuate the extent to which cortical thickness can increase with IV bisphosphonate therapy. It should be further noted that similar to osteogenesis imperfecta, prevention of long bone fractures is not guaranteed with IV bisphosphonate therapy in pediatric GIO, in part because cortical thickness will not increase when there is GC-induced linear growth retardation (109). The other reason that long bone fractures may persist despite IV bisphosphonate therapy, is that reductions in periosteal circumference, a feature of conditions with altered weight-bearing such as GC-naïve and GC-treated DMD, are not ameliorated with IV bisphosphonate therapy.

Together, the potential for vertebral body reshaping, and increases in cortical width (both growth-mediated), provide rationale for not withholding bisphosphonate therapy in children with GIO, despite the low bone turnover that is characteristic of the osteoporosis. For patients with attenuated linear growth, efforts should be made to restore normal growth using GC-sparing approaches if possible, and to recognize the limitations of anti-resorptive therapy when it is not possible to overcome the growth failure. In such cases, mitigation of incident VF and back pain are the main goals of IV bisphosphonate therapy.

### Longitudinal Growth Influences the Duration of Treatment

Once a decision has been made to provide bone protection in a GC-treated child, bisphosphonate therapy should be continued for as long as GC exposure persists at supra-physiological doses, and then for a period of time thereafter (1 year, provided the patient is showing signs of recovery from osteoporosis). A number of seminal observations unique to children have been responsible for this recommendation, as follows.

Following bisphosphonate discontinuation in patients with open epiphysis, and therefore ongoing endochondral bone formation, the newly formed bone adjacent to the growth plate is “treatment naïve,” and therefore lower in BMD. This creates a stress riser between higher density (i.e., recently treated), and lower density (i.e., untreated), bone (115). As a result, long bone metaphyseal fractures have been reported post-bisphosphonate discontinuation, at the interface between the treated and treatment-naïve bone, in patients with open epiphyses and persistent osteoporosis risk factors (116). In fact, metaphyseal fractures have even occurred *while receiving* cyclical IV bisphosphonate therapy, at the interface between the dense metaphyseal lines created at the time of each cycle, and the adjacent, treatment-naïve bone (117). This begs the question as to whether IV bisphosphonates should be administered with as short an interval between infusions as possible, a consideration that is challenged by patient burden due to frequent infusions. In any case, observations of fractures at the interface between treated and newly formed, treatment-naïve bone have led to the recommendation that bisphosphonates should be continued until the end of linear growth, as long as the risk factors for low BMD persist during this time.

For children with resolution of osteoporosis risk factors during growth (i.e., controlled inflammation, discontinuation of GC therapy, normal mobility), cessation of bisphosphonate therapy can be considered once the child has been fracture-free for at least 12 months after GC therapy has been stopped. This is provided, however, that previously fractured vertebral bodies have undergone reshaping in patients with residual growth potential. Cessation of bisphosphonate therapy is also contingent upon attainment of BMD Z-scores appropriate for height, and normal rates of age- and gender-matched bone mineral accrual (Figure 4). Re-introduction of bisphosphonate therapy may be required during growth if the prior risk factors for osteoporosis recur, and if the patient goes on to develop new bone fragility.

The rationale for the continuation of bisphosphonate therapy for 1 year following resolution of risk factors and signs of osteoporosis recovery, is based on a number of important observations. In children with leukemia, we observed a decline in annual VF and non-VF incidences by 4 years following diagnosis, but a slight increase in fracture rates between 4 and 5 years following leukemia diagnosis (with most children recently off chemotherapy at that time) (20). This suggested a period of relative fragility following chemotherapy discontinuation. A report over a decade ago in children with leukemia made a similar observation of increased fracture rates in the year following therapy cessation (118).

A study by Mostoufi-Moab et al. (119) went on to shed light on a possible explanation for the increased bone fragility in the year following GC cessation. Using peripheral quantitative computed tomography at the tibia in children who had recently completed leukemia therapy, it was shown that initial increases in cortical dimensions due to growth recovery were associated with declines in cortical BMD. A year later, cortical dimensions stabilized, which were followed by increases in cortical BMD (119). The authors speculated that the lag between growth-mediated increases in cortical dimensions, and subsequent increases in cortical BMD, may have resulted from the recovery time needed for newly-formed bone to undergo mineralization. This, in turn, might explain the period of relative bone fragility after completion of leukemia therapy that was observed in the two longitudinal studies from different countries (20, 118). Taken together, it appears that the year following resolution of risk factors, including cessation of GC therapy, may be a period of true bone fragility. Therefore, we recommend that bone protection continue for 1 year following GC cessation, to prevent fractures during this vulnerable phase of recovery.

## Unmet Needs That Drive Future Directions in the Management of Pediatric GIO

### The Need for a Pediatric GIO Treatment With a Convenient Route of Administration and Minimal Side Effects

The first-infusion side effects of IV bisphosphonate therapy, along with the inconvenience of the IV route, have spurred interest in alternative forms of anti-resorptive therapy. RANKL is an essential mediator of osteoclast formation, function and survival (120). Denosumab is a fully human, monoclonal antibody that targets RANKL to prevent the activation of RANK, thus inhibiting cortical and trabecular bone resorption, and increasing bone strength, without directly interacting with bone surfaces (121). Large studies in adults have shown that denosumab 60 mg every 6 months reduces hip, vertebral, and non-vertebral fracture risk without an increased frequency of adverse events, compared with placebo (122). Other adult studies have also confirmed that adverse events with denosumab are similar to an active comparator (oral alendronate), including the frequency and magnitude of hypocalcemia (122, 123). Denosumab is approved for osteoporotic men, and post-menopausal women, with a high risk for fracture; it is also approved for adult GIO. In children, the compassionate use of denosumab has been reported in a few studies of osteogenesis imperfecta (including osteogenesis imperfecta type VI, a sub-type that is not as responsive to IV bisphosphonate treatments as other forms) (124), giant cell tumors (125), aneurysmal bone cysts (126), and fibrous dysplasia (127). Currently, denosumab is being studied in children with osteoporosis, including GC-treated DMD, compared to standard-of-care IV zoledronic acid (NCT 02632916).

One concern with the use of denosumab is the “rebound phenomenon,” which includes loss of BMD, and an increase in VF, following denosumab administration in adults (128), and frank hypercalcemia-hypercalciuria in children (129). This phenomenon arises from exuberant skeletal resorption following reactivation of osteoclasts, presumably as the effect of the antibody wanes. In pediatric GIO, where bone turnover is invariably low, and linear growth is often attenuated, it is unclear whether this phenomenon will be a concern. On the other hand, rebound may theoretically occur when bone turnover increases at the time of puberty, or following GC cessation. These safety considerations merit careful study in clinical trials. Since RANKL is also implicated in the inflammatory pathway that contributes to muscle destruction in DMD (6), studies assessing the impact of denosumab on *muscle* strength in DMD are also of interest.

### Prevention of First-Ever Fractures Using Drugs That Are Anabolic to Bone, and Sequential Therapy

**Figure 12:**
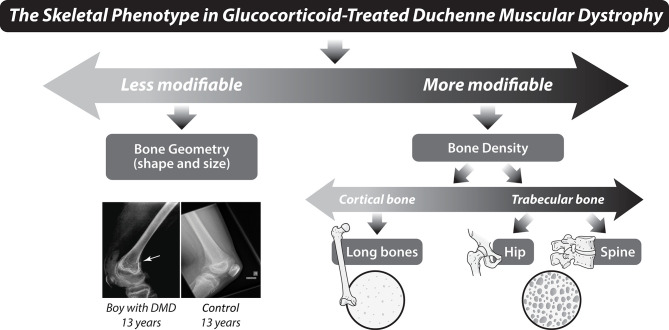
Aspects of the skeletal phenotype that are more or less modifiable by anti-resorptive osteoporosis therapy in glucocorticoid-treated Duchenne muscular dystrophy. Prevention of first fractures in glucocorticoid-treated, and in glucocorticoid-naïve, Duchenne muscular dystrophy is one of the greatest unmet needs in the pediatric osteoporosis field, at the current time. Note the arrow (left), pointing to a distal femur fracture in a boy with Duchenne muscular dystrophy, along with thin cortices, and a reduction in femur width, relative to the healthy control. DMD, Duchenne muscular dystrophy.

The BMD of trabecular-rich bone such as the spine is more readily modifiable by anti-resorptive therapy than cortical bone, because porous (spongy) bone has greater capacity to accommodate a bone density-altering therapy, compared with compact bone (Figure 12). In addition, while anti-resorptive therapy increases cortical width of long bones in children who are growing, the reductions in long bone periosteal circumference that are germane to diseases like DMD (Figure 12) are not modifiable by any therapy that acts only on endocortical and trabecular surfaces (such as anti-resorptive drugs); therefore, medications are needed that also target periosteal apposition.

As such, the door is decidedly open to novel therapies that are anabolic to bone, and which would be ideal in children with a need for prolonged GC therapy, with poor growth, and with reductions in periosteal circumference (the latter, whether due to myopathies as in DMD, or due to the periosteal apposition-limiting effects of prolonged GC). The overall goal of such an approach would be to prevent first-ever fractures in children with the greatest risk of bone fragility, and the least potential for recovery.

Teriparatide, recombinant human PTH (1–34), is approved by the United States Food and Drug Administration (FDA) for initial treatment of post-menopausal osteoporosis with a high risk of fracture, for patients who have failed prior osteoporosis therapy, and for adults with GC-associated osteoporosis (130). Teriparatide reduces the risk of VF, and non-VF, in post-menopausal women; the effect on fractures of the hip was inconclusive due to a low incidence of hip fractures in a large, randomized controlled trial (131). Overall, teriparatide positively affects spine BMD, but not BMD at the hip or forearm (131). Where children are concerned, this anabolic drug has an FDA black box warning against its use due to the development of osteosarcoma in growing rats treated at doses that were three to 50 times higher than human, adult equivalents (132). Subsequent studies in the same strain of rats found no evidence of malignancy with doses that were three times higher than the human equivalent (133).

BMD declines rapidly in the 12 months following teriparatide cessation, although fracture reductions persist for up to 2 years (134). Teriparatide, followed by alendronate, mitigates this loss (135). A recent case report of teriparatide in a 20 year old man with DMD described improvement in back pain due to VF after 6 months of teriparatide, plus increases in LS BMD, improvement in quality of life, and increases in suppressed bone biomarkers (136). These findings support further study of PTH in DMD post-epiphyseal fusion. On the other hand, the impact of PTH on bone is attenuated in adults when administered after bisphosphonate therapy (137). This may undermine its use in men with DMD who received bisphosphonates in childhood.

Sclerostin is a potent, negative regulator of bone formation, secreted by the osteocyte to inhibit the anabolic Wnt signaling pathway. Sclerostin antibody therapy releases the inhibition on sclerostin-mediated bone formation, and has been approved for women with post-menopausal osteoporosis (romosozumab). Mice treated with sclerostin antibody demonstrate not only increases in BMD and bone turnover markers, but show bone strength-enhancing changes in bone geometry (such as increases in periosteal circumference) that are not possible with anti-resorptive therapy (138). Sclerostin antibody-based drug trials for osteoporotic children with frequent fractures, low BMD, and reduced periosteal circumference are currently in the strategic development phase. In adults receiving anti-sclerostin antibody, bone formation returns to baseline by about 6 months after the first sub-cutaneous injection, and subsequent doses appear to have less of a beneficial effect on bone formation (139). As a result, it has been recommended to “seal in” the gains of sclerostin antibody treatment with a sequential therapy approach, using a long-acting anti-resorptive treatment (139, 140). These mechanisms of action will need to be considered in any future trial of sclerostin in pediatric GIO.

### There Is a Need for High Quality Intervention Studies That Acknowledge the Barriers to “Gold Standard” Clinical Trial Designs in Pediatric GIO

At the time of this writing, there are four drug trials listed on ClinicalTrials.gov targeting osteoporosis in GC-treated children (last accessed 28 June 2020). The first is a 1 year, international, phase 3, double-blind, randomized, controlled trial of intravenous zoledronic acid vs. intravenous placebo (both twice yearly) in children with GIO (NCT00799266, completed). The second is an extension to the aforementioned study, involving a 1 year, international, open-label extension of zoledronic acid twice yearly in children with GIO (NCT01197300, completed). The third trial is a 3 year, international, phase 3, double-blind, randomized, placebo-controlled, parallel-group study of denosumab in children with GIO (NCT03164928, recruiting). The fourth study registered with ClinicalTrials.gov is a 2 year, single-center, randomized, controlled, *pilot* study of 6 monthly intravenous zoledronic acid vs. 6 monthly sub-cutaneous denosumab in children with osteoporosis (NCT02632916, completed). This pilot study is sponsored by the author (LMW), in collaboration with the Children's Hospital of Eastern Ontario Research Institute. The study enrolled 10 children with osteoporosis, of whom eight had GC-treated DMD, and two had juvenile osteoporosis. The manuscripts for the three completed trials, examining the effect of zoledronic acid or denosumab on osteoporosis in GC-treated children, are currently in preparation for submission to peer-reviewed journals. Together, these trials will provide much-needed data on the use of anti-resorptive therapy in pediatric GIO.

While we eagerly await the results of these trials, it should be recognized that if medications to treat pediatric GIO are ever to achieve health authority-endorsed labeling on sufficient numbers of patients, pediatric trials may need to be adjudicated differently than adult osteoporosis studies. The pediatric approach must not compromise on safety data, but should acknowledge the debilitating consequences of pediatric GIO, and the need for guidance on the treatment of GIO in childhood, despite the formidable logistical challenges of studies in children with uncommon conditions. To this end, pragmatic study designs may be needed that provide reasonable proof of efficacy, including biomarkers to demonstrate the anticipated biology of the drug, surrogates of bone strength (e.g., BMD and bone geometry), patient-reported outcomes (e.g., pain, quality of life), functional studies (e.g., mobility), and careful fracture phenotyping (including vertebral fractures). Methodologically sound patient registries, and natural history comparator studies, may help overcome barriers in this context. Ultimately, innovative drug studies that maneuver the challenges of pediatric GIO trials are needed, in order to avoid uninformed off-label prescription in a well-intentioned effort to help a child with GIO.

## Author Contributions

The author confirms being the sole contributor of this work and has approved it for publication.

## Conflict of Interest

LW has participated in clinical trials with ReveraGen BioPharma, PTC Therapeutics, Catabasis Pharmaceuticals, Novartis, Ultragenyx, and Amgen. LW has also received unrestricted educational grants from Alexion and Ultragenyx, and consulting fees from Ipsen, Ultragenyx, PTC Therapeutics, Novartis, and Amgen, with funds to LW's institution.
